# Are Grignard Reactions in Deep Eutectic Solvents Interface‐Driven?

**DOI:** 10.1002/anie.202513649

**Published:** 2025-09-01

**Authors:** Iva Manasi, Marco Bortoli, Daniel T. Bowron, Mario Campana, Oliver S. Hammond, Thomas F. Headen, Jake Hooton, Eva Hevia, Michele Cascella, Odile Eisenstein, Karen J. Edler

**Affiliations:** ^1^ Department of Physics University of Bristol, Tyndall Avenue Bristol BS8 1TL United Kingdom; ^2^ Department of Chemistry University of Bath Claverton Down Bath BA2 7AX United Kingdom; ^3^ Department of Chemistry and Hylleraas Centre for Quantum Molecular Sciences University of Oslo PO Box 1033 Blindern 0315 Oslo Norway; ^4^ ISIS Neutron and Muon Source Rutherford Appleton Laboratory Oxford OX11 0QX United Kingdom; ^5^ European Spallation Source ERIC Data Management & Software Centre Asmussens Allé 305 Kongens Lyngby 2800 Denmark; ^6^ Department für Chemie Biochemie und Pharmazie Universität Bern Freiestrasse 3 Bern 3012 Switzerland; ^7^ ICGM Université de Montpellier, CNRS, ENSCM 1919 Route de Mende Montpellier 34293 France; ^8^ Department of Chemistry, Centre for Analysis and Synthesis (CAS), Lund University Lund 221 00 Sweden

**Keywords:** Biphasic systems, Green chemistry, Interfacial reactions, Non‐volatile solvents, Solvophobic effect

## Abstract

Due to their high reactivity, organolithium and organomagnesium addition to ketones is usually performed under inert atmosphere at low temperature. Recent work has shown that, by dissolving the substrate in deep eutectic solvents (DES), these processes can be carried out on the benchtop, in air at room temperature. Surprisingly, the organometallic reagent, added to the DES from an organic solution, works in these conditions and gives better yields than in the standard setup. Here, we investigated acetophenone in a (1:2) choline chloride:glycerol (ChCl:Gly) DES solution by experimental liquid diffraction, neutron reflectometry, NMR, interfacial tension measurements, and by computational modelling. Our data show that this DES is a poor solvent for the ketone and promotes its accumulation at the surface of the liquid or its escape into the organic solvent. Molecular dynamics simulations of Grignard reagent *i*‐PrMgCl in the (ChCl:Gly)/tetrahydrofuran biphasic system indicate also preference for its localisation at the interface. These results pinpoint why this combination of solvents promote the reaction, require stirring, and accounts for the lack of rapid decomposition of the organometallic reagents.

## Graphical abstract

Experimental and molecular dynamics studies of acetophenone, alongside a computational analysis of *i*‐PrMgCl in a biphasic ChCl:2Gly DES/THF solution, offer valuable insights into the remarkable efficiency of the Grignard reaction under these conditions. The poorly soluble acetophenone interacts with the Grignard reagent at the interface of the two solvents, enhancing the probability of effective contact between the reagents.

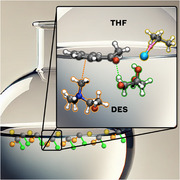

## Introduction

Deep eutectic solvents (DES) are room‐temperature liquids formed by molecular mixtures with a lower melting point at the eutectic composition relative to that predicted by ideal mixing.^[^ ^1^, ^2^, ^3^ ^]^ They have a “liquid window” around the eutectic molar fractions, which makes them attractive and applicable as general‐purpose solvents.^[^ ^3^, ^4^ ^]^ DES can show high thermal stability, non‐flammability and low vapour pressure, therefore low volatility.^[^ ^1^ ^]^ Typical DES components (e.g., choline chloride, urea, natural carboxylic acids, amino acids and carbohydrates, polyalcohols, etc.) can be obtained from renewable sources and are gaining interest as greener and more environmentally benign solvents to replace toxic, volatile organic solvents in electrochemistry and organic synthesis, as well as in preparation of nanoparticles^[^ ^5^, ^6^ ^]^ and dissolution of biomolecules.^[^ ^7^ ^]^

DES have recently been shown to contribute uniquely to reaction chemistry and syntheses for a range of components.^[^ ^8^, ^9^, ^10^, ^11^ ^]^ Organolithium and organomagnesium (Grignard) additions to ketones are one of the most versatile and fundamental methods to generate new C─C bonds, allowing the formation of tertiary alcohols.^[^ ^12^, ^13^, ^14^, ^15^, ^16^, ^17^ ^]^ Traditionally, these reagents need to be used under inert atmosphere conditions, with dry and degassed organic solvents, and strict control of the temperature. Breaking new ground in this area, in 2014 Vidal et al.^[^ ^14^ ^]^ reported the chemoselective alkylation of ketones by RMgX and RLi reagents in water‐containing DES exposed to air, at room temperature—conditions normally incompatible with polar organometallics. Yet, in some DES, these reactions resulted in improved yields and better selectivity than when using conventional inert atmosphere protocols. Moreover, they were less energy intensive, markedly more environmentally friendly, and safer. In particular, using eutectic mixtures, choline chloride:glycerol (ChCl:Gly) and choline chloride:water (ChCl:W) solvents uncovered a unique activating effect favouring the formation of the alkylation product over the competing hydrolysis of the organometallic reagent. Remarkably, these reactions, in which the Grignard reagent is added in an ethereal solvent, are very fast and compatible with the presence of air.

Specifically, the reaction is successfully carried out by adding an organic solution of the organometallic reagent into a solution of substrate in the DES while rapidly stirring.^[^ ^18^, ^19^ ^]^ Interestingly, stirring is key in order to obtain good yields for the alkylation reaction—if the reaction is performed in the absence of stirring the relevant tertiary alcohol is obtained in modest yields. Reactivity studies have also shown that if the organometallic reagent is directly mixed with the DES, reaction with DES components occurs over time; if substrate is added more than 2 min later, no alkylation product is observed. This suggests that the organometallic reagent has decomposed in contact with the DES. Thus, under appropriate experimental conditions, organomagnesium or organolithium react preferentially with the substrates rather than the DES components, despite the latter containing OH groups that are known to react rapidly with such reagents. On the contrary, attempts to carry out the same reactions in glycerol or water alone were not successful, demonstrating the importance of the DES to facilitate these C─C bond forming processes.^[^ ^20^ ^]^ Similar effects occurred in chemoselective nucleophilic arylation/alkylations of non‐activated imines.^[^ ^20^ ^]^ Despite these synthetic advances, the exact role of the DES still remains unclear, although previous experimental studies have hinted at some type of substrate activation involving the ketone and the ammonium salt choline chloride, a component of the DES employed. Moreover, while this setup significantly reduces the formation of by‐products, it is not systematically advantageous for all substrates or Grignard reagents.

**Figure 1:**
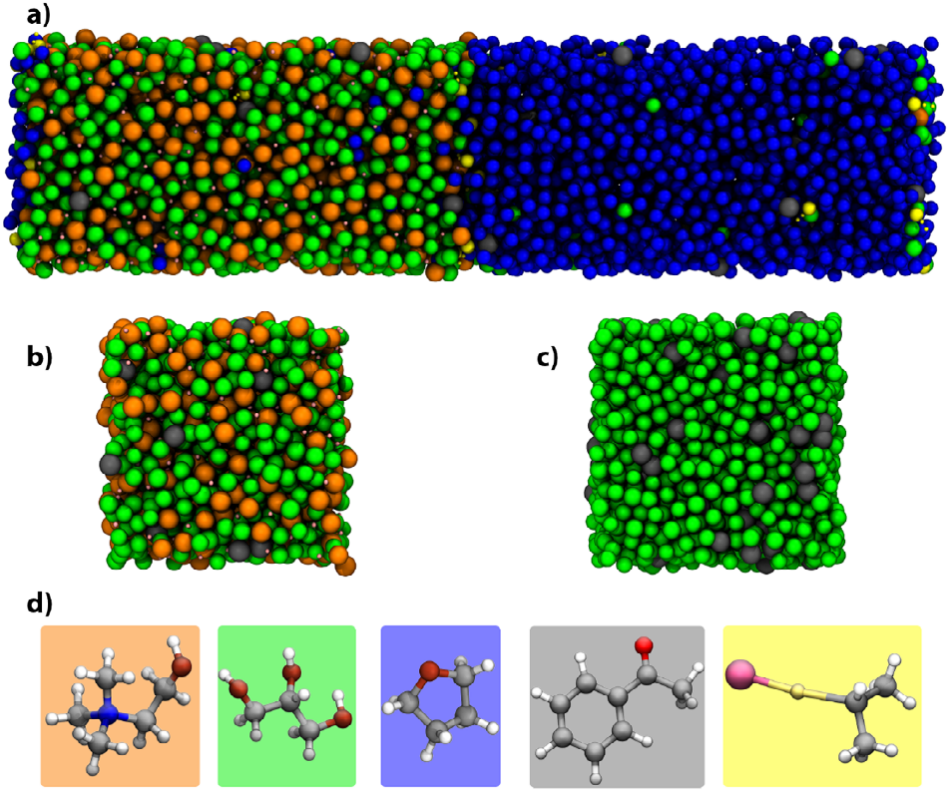
Model depiction of the investigated systems: a) ChCl:Gly DES biphase system with THF, containing AcPh and *i*‐PrMgCl, b) ChCl:Gly with AcPh, c) pure Gly with AcPh and d) atomistic structure of the systems' components. In the image, molecules are represented as beads located at their centre of mass. Colour code: Ch^+^: orange; Gly: green; THF: blue; AcPh: grey; *i*‐PrMgCl: yellow.

The aim of this work is to reveal the physical and chemical drivers behind these remarkable experimental conditions, thus enabling improvements toward better yields over a wider range of substrates. For this, we have obtained experimental structural characterisation by wide‐angle neutron scattering, NMR, tensiometry, and neutron reflectivity, of a choline chloride:glycerol (1:2) DES solution containing acetophenone (AcPh). In addition, we provide a computational model of this solution,^[^ ^21^ ^]^ also in phase co‐existence with isopropyl magnesium chloride (*i*‐PrMgCl) in tetrahydrofuran (THF), thus proposing for the first time a detailed molecular picture of this reaction mixture (Figure 1). Our combined experimental and computational results point at a fundamental role of the liquid–liquid interface, favoured by the mechanical stirring, in promoting the reaction. This setting was selected because it is associated with the best observed yield and selectivity.^[^ ^14^ ^]^

## Results and Discussion

### AcPh Prefers Dissolving into Glycerol Than DES

Liquid neutron diffraction data was collected at 25 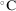 from 1:2 molar ratio ChCl:Gly without and with the addition of AcPh and for glycerol with AcPh. Isotopic substitution of deuterium for hydrogen in choline chloride, glycerol and AcPh allowed 6 different solutions to be measured for the ChCl:Gly DES, eight different solutions were measured with added AcPh and four different solutions of glycerol with added AcPh (see details in Section S3). The data was modelled using a Monte Carlo‐derived method, Empirical Potential Structure Refinement (EPSR),^[^ ^22^, ^23^ ^]^ (Figures S4–S6) and compared to molecular dynamics simulations.

#### Pure ChCl:Gly (1:2)

Initially, we benchmarked the experimental and modelling setup by characterising ChCl:Gly (1:2) DES, and comparing it to literature data.

**Figure 2:**
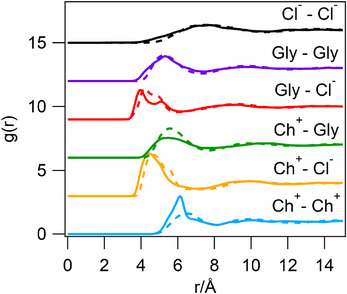
Molecular RDFs for ChCl:Gly DES components from EPSR (solid lines) and MD simulations (dashed lines). The computed RDFs are calculated between representative molecular centroids. The plots are stacked along the *y*‐axis for clarity.

Molecular radial distribution functions (RDFs) at 25 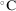, between the central atoms of the components ${\mathrm{Ch}}^{+}$, ${\mathrm{Cl}}^{-}$ and Gly, calculated using both the EPSR model from the experiment, and from MD simulations, report peaks in very good mutual agreement (Figure 2, Table 1). They are comparable to those previously reported for the ChCl:Gly DES measured at 60 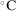 by Turner and Holbrey (Tables 1 and S2).^[^ ^24^ ^]^ Our data show that the most likely associations feature Gly–Gly interactions, with a coordination number of 7.7±2.3, and ${\mathrm{Ch}}^{+}$–Gly with a coordination number of 7.6±2.3, which is explicable by the fact that glycerol is more abundant and has more H‐bonding sites. This is consistent with the structure at 60 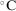 reported by Turner and Holbrey who suggested an extensive and persistent, homo‐molecular glycerol hydrogen bonding network for ChCl:Gly DES that is key to its formation.^[^ ^24^ ^]^

**Table 1:** The position of the first peak (*r*, Å) in the radial distribution functions (RDFs) and the coordination numbers (CN) for the various molecules calculated using the COORD routine in EPSR and from MD simulations for the ChCl:Gly DES. The data from Turner and Holbrey^[^ ^24^ ^]^ is also given for comparison. Uncertainties for the coordination numbers from ESPR and MD models are the standard deviation of the data collected during the corresponding simulations.

	Exp. 25 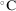		MD		Exp. 60 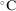	
	EPSR (this work)		(this work)		(Ref. [24])	
RDF	Peak	CN	Peak	CN	Peak	CN
Gly−Gly	5.3	7.7 ± 2.3	5.3	6.9 ± 1.8	5.3	7.5 ± 1.7
Gly−Cl^−^	4.0	1.9 ± 0.9	4.3	2.2 ± 0.9	4.1	2.0 ± 1.0
Gly−Ch^+^	5.6	7.6 ± 2.3	5.6	9.1 ± 1.2	5.9	8.4 ± 1.6
Ch^+^−Cl^−^	4.5	3.7 ± 1.2	4.7	4.0 ± 1.0	4.1	3.1 ± 1.1
Ch^+^−Ch^+^	6.2	3.5 ± 1.5	6.4	4.2 ± 1.5	6.5	2.5 ± 0.9
Cl^−^−Cl^−^	7.2	9.7 ± 2.3	7.7	11.3 ± 1.3	7.1	10.1 ± 2.5

A similar structure has also been reported by Faraone et al.^[^ ^25^ ^]^ who suggested that the DES comprises a glycerol H‐bonding network with interstitial choline ions. Nonetheless, our data at 25 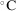 show that the peaks associated with ${\mathrm{Ch}}^{+}$–Gly and ${\mathrm{Ch}}^{+}$–${\mathrm{Ch}}^{+}$, are at shorter distances compared to Turner and Holbrey's measurements on the same system at 60 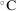 ^[^ ^24^ ^]^ (Table 1), while peaks associated with all other molecules are at similar distances. Although based only on observations made at two state points, 25 and 60 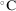, this suggests the ${\mathrm{Ch}}^{+}$ interactions are responsible for systematic thermal expansion in this comparative temperature range. Further studies would be necessary if this phenomenon is to be investigated in detail.

#### Solutions of AcPh in ChCl:Gly (1:2), and in Gly

**Figure 3:**
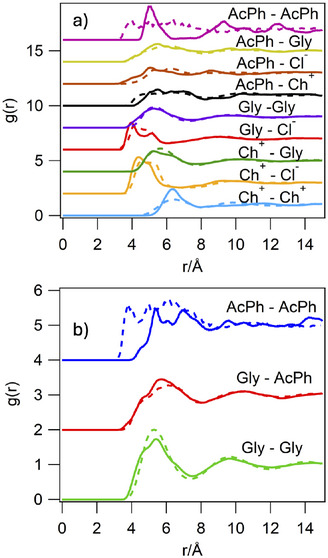
Molecular RDFs for a solution of DES with AcPh a), and for a solution of glycerol and AcPh b). Molecular RDFs from EPSR are shown by the solid lines, results from MD simulations are shown by the dashed lines. The computed RDFs are calculated between representative molecular centroids. The plots are stacked along the *y*‐axis for clarity.

NMR measurements revealed that AcPh had an unexpectedly low solubility in ChCl:Gly (0.2 mmol $g^{-1}$ or 2.2 mol%, ca. 2.4 wt%; Figure S1), compared to that in pure glycerol (0.75 mmol $g^{-1}$ or 6.7 mol%, ca. 9 wt%; Figure S2). To gain insight into the different molecular interactions in these two solvents, the RDFs for the two solutions were characterised by both EPSR and MD. As visible in Figure 3a, compared to the pure solvent, addition of AcPh to ChCl:Gly does not produce meaningful changes in the peak positions or shapes of the RDFs between the solvent components (Table 2). This is true also for the AcPh solution in Gly, which shows the Gly–Gly peak locating at 5.4 Å, again in agreement with that previously reported by Towey et al. for pure glycerol.^[^ ^26^ ^]^ Considering that the addition of AcPh does not modify significantly the density of the solvents, we conclude that AcPh does not alter the associations within the solvent. The AcPh–AcPh RDF shows higher noise due to the small number of AcPh molecules in the simulation. Overall, due to the higher concentration, we observe more numerous AcPh–AcPh associations in pure Gly than in ChCl:Gly DES (Table 2). This pair is the one for which we identify a discrepancy between the MD and EPSR models both in ChCl:Gly and in glycerol. The MD model seemingly overestimates the stability of AcPh–AcPh stacking geometries compared to T‐shaped organisations, yielding shorter AcPh–AcPh distances.

**Table 2:** The position of the first peak (*r*, Å) in the radial distribution functions (RDFs) and the coordination numbers (CN) for the various molecules calculated using the COORD routine in EPSR and from MD simulations for a 0.2 mmol $g^{-1}$ (6.7 mol%) solution of AcPh in ChCl:Gly DES and for a 0.75 mmol $g^{-1}$ solution of AcPh in pure Gly. Uncertainties for the coordination numbers from ESPR and MD models are the standard deviation of the data collected during the corresponding simulations.

	DES				Gly			
	EPSR		MD		EPSR		MD	
RDF	Peak	CN	Peak	CN	Peak	CN	Peak	CN
Gly–Gly	5.3	7.2 ± 2.1	5.3	6.7 ± 1.9	5.4	8.3 ± 2.3	5.3	12.5 ± 1.7
Gly–Cl^−^	4.0	1.9 ± 0.9	4.3	2.2 ± 0.9	–	–		
Gly–Ch^+^	5.3	7.8 ± 2.2	5.6	8.8 ± 1.4	–	–		
Ch^+^–Cl^−^	4.4	3.4 ± 1.2	4.7	3.8 ± 0.9	–	–		
Ch^+^–Ch^+^	6.3	3.9 ± 1.6	6.4	4.0 ± 1.3	–	–		
Cl^−^–Cl^−^	7.5	9.1 ± 2.2	7.7	11.0 ± 1.8	–	–		
AcPh–Ch^+^	5.5	3.7 ± 1.4	6.4	4.9 ± 1.2	–	–		
AcPh–Cl^−^	5.1	2.4 ± 1.2	6.2	4.2 ± 1.2	–	–		
AcPh–Gly	5.5	7.6 ± 2.1	6.1	9.4 ± 1.2	5.4	14.0 ± 2.0	6.2	13.7 ± 2.4
AcPh–AcPh	5.0	0.2 ± 0.4	4.0	0.04 ± 0.2	5.7	1.2 ± 0.9	4.0	0.14 ± 0.03

**Figure 4:**
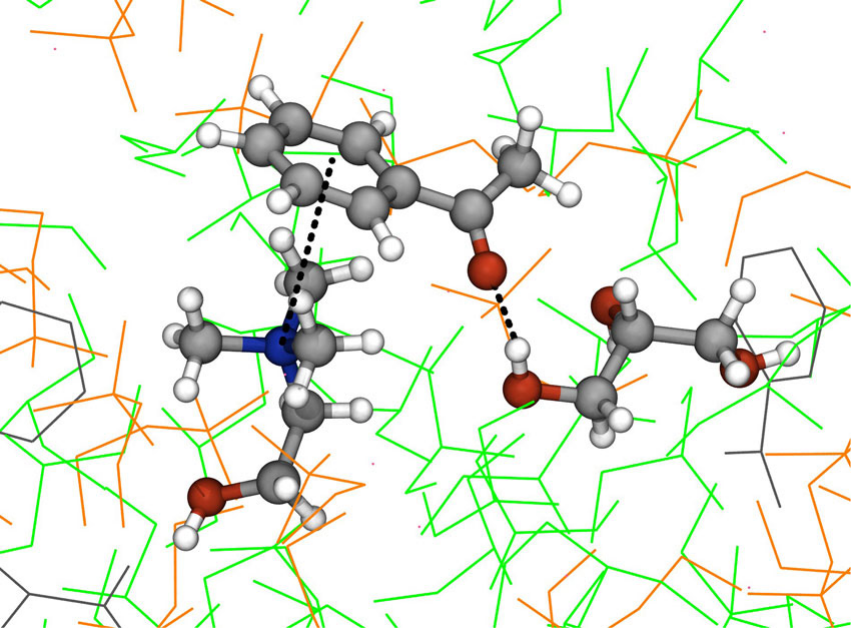
Non‐covalent interactions between AcPh, Gly and Ch^+^ at DES interface, identified during MD. H‐bonding with Gly and π–cation interactions with Ch^+^ are evidenced by dotted lines. Surrounding DES is represented in lines, with colour code: Ch^+^: orange; Gly: green; AcPh: grey.

The position of the AcPh–Gly peaks are similar in both solutions, with a first maximum at ∼5.5 Å and a secondary peak at 6.7 Å (Figure 3). Closer inspection of the corresponding all‐atom detailed molecular structures reveals that these peaks are associated with the two possible AcPh–Gly H‐bond modes; through the hydroxyl group attached to the central C atom, or through the hydroxyl groups at the terminal C atoms (Figure 4). The first peak also comprises vdW‐dominated hetero‐dimers between glycerol and the extended π‐electron cloud of AcPh.

In ChCl:Gly, the Ch^+^–AcPh interactions are found at a similar distance with a peak at 5.5 Å and a broad distribution around 6.3 Å. Also, in this case, binding is dominated by H‐bonding of the AcPh with the hydroxyl group of Ch^+^, and cation–π stacking of the Ch^+^ head with the extended π system of AcPh (Figure 4). Remarkably, however, in ChCl:Gly, the strongest associations for AcPh are observed with Gly, with a coordination number of 7.6±2.1 followed by choline (coordination number 3.7±1.4) and then chloride (coordination number 2.4±1.2). These interactions indicate interstitial accommodation of AcPh in the glycerol H‐bonding network of the DES, with AcPh strongly interacting with Gly and having less preferred interactions with Ch^+^ and ${\mathrm{Cl}}^{-}$.

The H‐bond network was calculated from MD simulations and showed a very similar picture for pure ChCl:Gly and the 0.2 mmol $g^{-1}$ solution with AcPh (Table S6). The most noticeable effect of the addition of AcPh was a slight decrease in the number of H‐bonds between Gly molecules confirming the preferential interaction of AcPh with Gly. Moreover, the number of Gly–AcPh H‐bonds in the DES (0.27±0.09) is found to be much lower than that in pure Gly (0.82±0.14) confirming that in the DES Gly–Ch^+^ interactions are preferred over those between Gly and the solute.

The self‐diffusion coefficients of the different components of the ChCl:Gly DES were calculated from MD with and without AcPh. Both systems show similar results (see Table S7), corroborating the idea that the structure of the DES is not perturbed by the presence of AcPh. Ch^+^ had the largest difference and showed a higher diffusion coefficient in the AcPh solution.

From these results it became clear that there are no special intermolecular interactions in these solutions that might be “activating” the AcPh substrate during the Grignard reactions. AcPh has limited solubility in the ChCl:Gly DES, and there are no distinctly different interactions of glycerol and choline with AcPh in the DES, compared to those in pure glycerol. Yet, glycerol alone was not an effective solvent for organomagnesium or organolithium additions in synthetic experiments, at least to imines.^[^ ^20^ ^]^

### Liquid/Air Interfaces Show Enhanced Surface Activity of AcPh in DES

**Figure 5:**
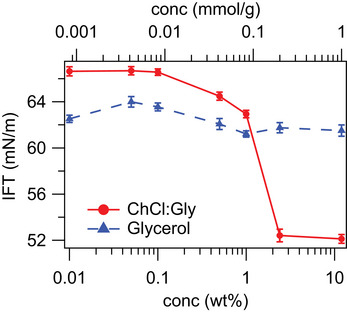
Interfacial tension measured using the drop‐shape‐analysis method with varying AcPh concentration in the solvent at the (ChCl:Gly)/air interface (red) and glycerol/air interface (blue). The data reported is the average value of the measurements with the standard error. The lines are drawn as guide to the eye.

Given the poor bulk solubility of acetophenone in the ChCl:Gly DES, and its lack of strong interactions with DES components, we hypothesised that perhaps a solvophobic effect instead causes the acetophenone to concentrate at the DES–organic solvent interface. To test the surface activity hypothesis for AcPh, surface tension measurements were made for varying concentrations (0.0008--1 mmol $g^{-1}$ or 0.01--12 wt%) of AcPh in DES and AcPh in glycerol solutions at the air interface (Figure 5). The addition of AcPh substantially reduced the surface tension at the ChCl:Gly–air interface to 52.4±0.5 mN $m^{-1}$, compared to the pure ChCl:Gly (1:2) surface tension of 66.9±0.2 mN $m^{-1}$. The interfacial tension continued to decrease with increasing concentration until ca. 2 wt% AcPh, indicating saturation of the surface activity occurring at around 0.2 mmol $g^{-1}$, corresponding with the limiting solubility measured by NMR above.

Similarly, MD simulations reported a decrease in surface tension, from 65.0±2.4 to 60.3±0.7 mN $m^{-1}$ in the case of the 0.2 mmol $g^{-1}$ solution, with clearly visible accumulation of AcPh at the interface. Conversely, experimental measurements of interfacial tension for AcPh in pure glycerol (Figure 5) showed little meaningful change (63.2±0.2 mN $m^{-1}$ for pure glycerol and 61.5±0.5 mN $m^{-1}$ for AcPh concentrations as high as 1 mmol $g^{-1}$) indicating no accumulation of AcPh at the glycerol/air interface, confirming again the different behavior of AcPh in DES and pure glycerol.

### AcPh is Surface Active Also at DES/Organic–Solid Interfaces

Given the well‐known experimental difficulties of directly measuring structures at liquid–liquid interfaces, we therefore investigated a model liquid hydrophobic interface to mimic the DES–organic interface.^[^ ^27^, ^28^ ^]^ Specifically, we employed silicon blocks coated with octadecyltrichlorosilane (OTS) to create a liquid‐like hydrocarbon layer,^[^ ^29^ ^]^ which was then exposed to the DES solution in a solid–liquid flow cell, and the interfacial accumulation of AcPh was characterised using neutron reflectometry (see details in Section S4).

**Figure 6:**
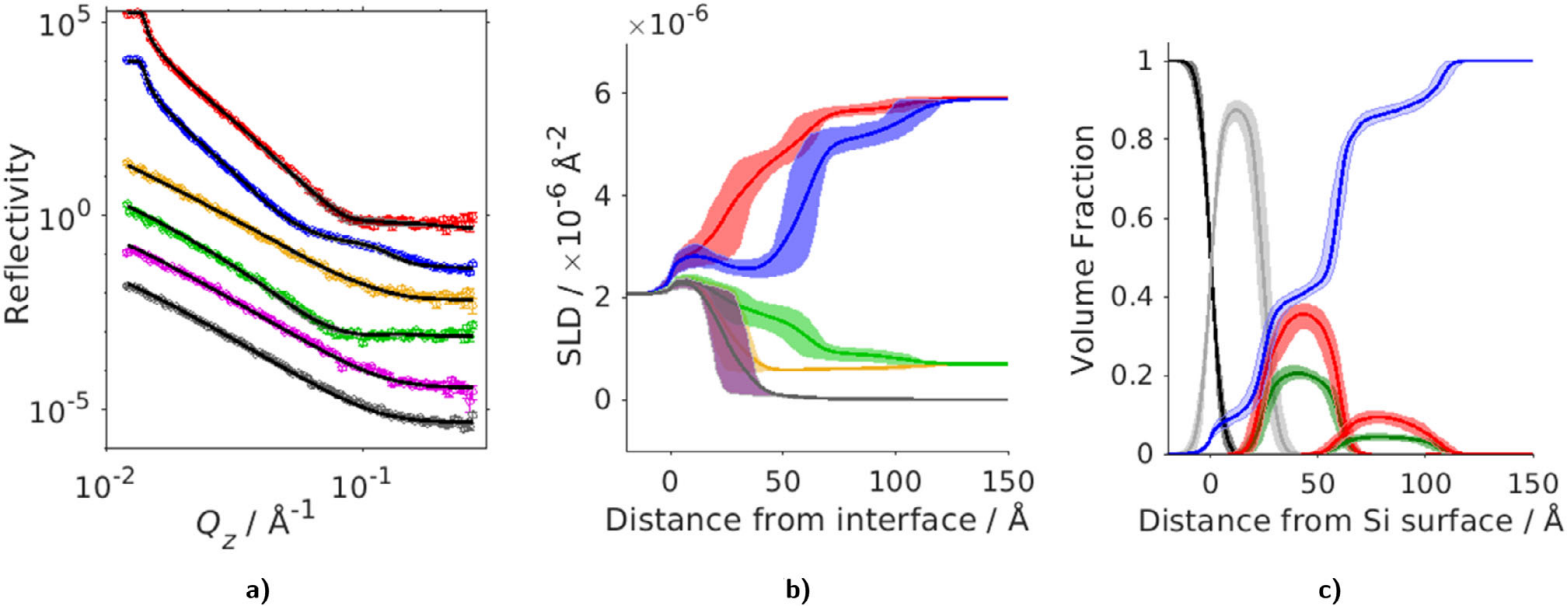
Neutron reflectivity measurements and fits for the ChCl:Gly/OTS interface without and with added AcPh. a) Reflectivity curves and fitted reflectivity profiles, with a 65% confidence interval, from OTS/air (grey and magenta), OTS/d‐DES (red) and OTS/h‐DES (yellow), OTS/d‐DES+h‐AcPh (blue) and OTS/h‐DES+d‐AcPh (green) interface. b) The corresponding SLD profiles with a shaded region showing the 65% confidence interval on the fit parameters. c) The volume fraction distribution of all the components across the interface: silicon (black), silicon oxide (grey), OTS (green), AcPh (red) and the DES (blue). The shaded region represents the 65% confidence interval on the parameters.

Pure ChCl:Gly DES and a solution of AcPh in the ChCl:Gly were flowed through the cell and the interfacial structure was measured. The interface was modelled as a layered structure on the silicon oxide‐capped silicon substrate, comprising an OTS layer solvated by the DES for the pure DES case and an OTS and AcPh layer solvated by the DES for the solution of DES with AcPh. The measured reflectivity profiles and fits of the DES/OTS interface with and without added AcPh, along with the scattering length density (SLD) profile of the fitted interfacial layered structure for the various layers, are shown in Figure 6 (for additional insight into the fitting, see Figure S9 and Table S5).

The neutron reflectivity measurements show that for the DES‐only solution, the interface has a first OTS layer of thickness 32 Å which is swollen by substantial amounts of the DES (OTS volume fraction $\phi_{\text{OTS}}=0.2$, DES volume fraction $\phi_{\text{DES}}=0.8$). A second, very low density OTS layer, 41 Å with $\phi_{\text{OTS}}=0.05$ and $\phi_{\text{DES}}=0.95$, is also visible, likely due to a few silane clusters, formed in solution during initial deposition of the OTS monolayer, which adhered on top of the first OTS layer during monolayer preparation. Beyond this, pure bulk DES is observed (see also Figures S8 and S10). In the DES solution containing AcPh, the AcPh accumulates from solution into the first OTS layer, displacing some of the DES. The near‐surface layer now becomes $\phi_{\text{OTS}}=0.2$, $\phi_{\text{AcPh}}=0.4$ and $\phi_{\text{DES}}=0.4$. AcPh also displaces some of the solvent in the second layer ($\phi_{\text{OTS}}=0.05$, $\phi_{\text{AcPh}}=0.1$ and $\phi_{\text{DES}}=0.85$). This experiment provides further evidence that AcPh preferentially adsorbs into the interface between the bulk DES and a hydrophobic liquid‐like layer, as well as forming a surface excess at the air–DES interface.

### Computer Simulations of Substrate in Biphasic DES/THF Inform on the Localisation of Reactive Species

A liquid–liquid (ChCl:Gly)/THF system was simulated with classical MD (Figure 1). AcPh was randomly added to the system in two independent setups at 0.2 and 1.0 mmol $g^{-1}$ concentration (details in Section S5).

**Figure 7:**
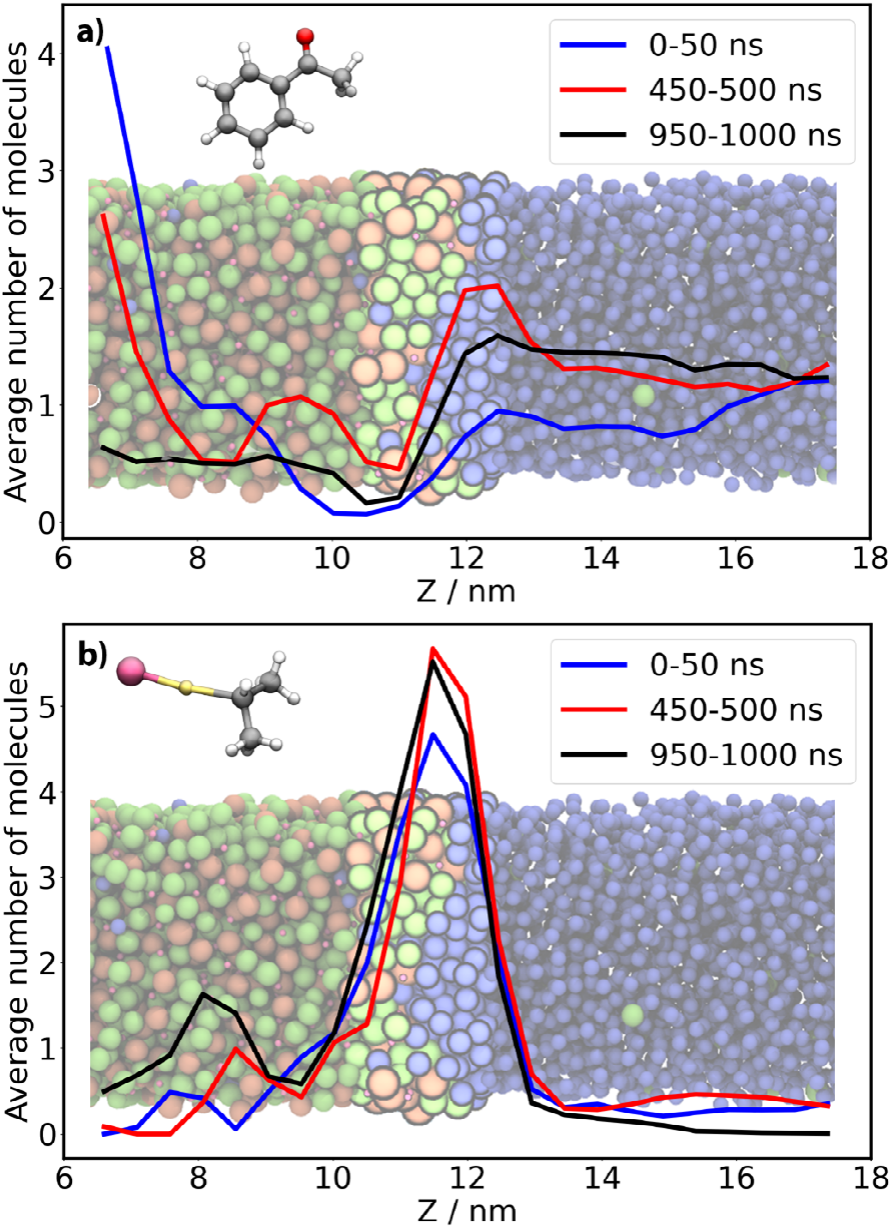
Average number of AcPh at the experimental 0.2 mmol $g^{-1}$ concentration a), and of *i*‐PrMgCl molecules b) along the *Z* coordinate in the biphase (ChCl:Gly)/THF system at different simulation times.

Noticeably, for both concentrations of AcPh, progressing along the simulation time, an increasing number of AcPh molecules migrated into the THF region (Figure 7a). Another indication of the AcPh transfer was provided by the RDFs between the centres of mass of AcPh and THF at different simulation times (Figure S11), which, for both concentrations, returned an average solvation number of ∼10 THF molecules around AcPh in the first 50 ns of the simulation that increased to ∼12 molecules after 450--500 ns. The qualitative profile of the RDFs remained unaltered, whereas only the height of their peaks increased at later stages of simulations, meaning that the increase in the coordination number is simply due to more AcPh being in bulk THF, rather than to a change in the interactions between the two molecules.

Quantitative analysis of the free energy of the transfer of AcPh from the DES to the organic phase was conducted employing umbrella sampling simulations (Section S5). We found that the free energy of AcPh is higher in DES, with a net decrease through the interfacial region, reaching a global shallow minimum value at the first layer of the THF phase (∼ ‐1 kJ ${\mathrm{mol}}^{-1}$ with respect to bulk THF). This free energy profile suggests that the AcPh molecules escaping the DES phase remain loosely bound to the interface, and can easily further diffuse into the bulk organic liquid. Overall, the preference of AcPh for the organic phase with respect to the ChCl:Gly (1:2) DES is approximately 7.5 kJ ${\mathrm{mol}}^{-1}$ (see Figure S12). At the (ChCl:Gly)/THF interface, and before entering into the bulk organic liquid, AcPh retains the same type of interactions with the DES as characterised in the bulk (π–cation interactions with ${\mathrm{Ch}}^{+}$ and H‐bonding with Gly, see Figure 4).

Finally, we simulated a (ChCl:Gly)/THF system containing both AcPh and the Grignard reagent *i*‐PrMgCl at a concentration of 0.2 mmol $g^{-1}$. The two solutes were initially located in DES and THF, respectively. Again, AcPh was seen to move towards the organic phase from the bulk DES, with transient interfacial accumulation in the first 500 ns of simulation time. On the contrary, the MD data reported clear enrichment of the organometallic species at the interfacial region already after the first 50 ns (Figure 7b).

### Role of the Biphasic Liquid/Liquid Setup for the Grignard Reaction

To understand the improvements that this DES, formed from a 1:2 mixture of ChCl and Gly, offers for organometallic chemistry^[^ ^30^ ^]^ in light of this work, it is advantageous to summarise the unusual experimental conditions that are associated with its use. First, a DES solution containing the substrate (for example acetophenone) is prepared. Then, a second solution of the Grignard in an organic solvent (for example, THF) is added to the first one. Rapid stirring while adding the second solution into the first is mandatory for the highest yields; without stirring, yields are considerably reduced or eventually lost.

NMR measurements show that AcPh has relatively low solubility in the ChCl:Gly DES (see Supporting Information). Neutron scattering and MD show that adding AcPh does not modify the structural features of ChCl:Gly. Furthermore, the same EPSR and MD data show that the network of H‐bonds that stabilises AcPh in pure Gly is diminished in the DES, in favour of preferential glycerol–choline interactions, making the ChCl:Gly mixture a poorer solvent for AcPh than pure glycerol. Finally, no specific interaction between AcPh and Ch^+^ could suggest an activation of the substrate by choline, as was previously considered to explain the improved reactivity.^[^ ^14^, ^20^ ^]^

The weak solvation properties of ChCl:Gly result in AcPh being pushed away from the bulk of the DES towards the surface in case of DES/air and model DES/hydrocarbon interfaces. In the case of a (ChCl:Gly)/THF biphase, our MD simulations predict that AcPh leaves the DES to enter the more favourable organic phase. The same simulations predict that *i*‐PrMgCl, initially located in bulk THF, migrates and accumulates at the (ChCl:Gly)/THF interface, without crossing into the DES phase. This preference for going to the interface is due to the attraction of the polar Mg‐X bond toward the polar DES, while the more hydrophobic organic group R group prevents crossing, the Grignard reagent thus acting as an amphiphilic compound in this system.

These findings point at a straightforward interpretation of the efficiency of the Grignard reaction in ChCl:Gly. In the biphasic solvents, the organic substrate prefers to leave DES to enter the organic phase. By doing so, it has to cross the interface where the Grignard reagent accumulates. This forced encounter in a confined space favours the reaction between the two species. The interface offers also a protection against the destruction of the Grignard by the protons of the DES. At the interface, the two solvents and the substrate mix, resulting in a media which, due to the diminished concentration of protic species, is more protective to the Grignard reagent. Thus, in place of being hydrolysed, the Grignard reacts with the organic substrate. One can also understand the key role of rapid stirring. By stirring, the organic phase forms small bubbles in the DES, increasing the proportion of the interface relative to the bulk organic phase, and therefore, increasing the probability of the two reagents to meet and then to react. The need for stirring is not at all unique to this specific reaction—the anionic polymerisation of styrene in DES promoted by BuLi requires ultrasound to create a fine dispersion of the olefins in the same DES.^[^ ^18^ ^]^ Thus, also in this case, significant expansion of the interface area is mandatory for the process to take place. This analogy reinforces the importance of maximising interfacial contact between the organic and DES phases for efficient reactivity.

Our study was limited to one DES of type III (quaternary ammonium salt and hydrogen bond donor). In fact, the diversity of DES is large,^[^ ^1^ ^]^ leaving open the question of the generality of this mechanism. Although further tests are needed to assess this, emerging literature suggests that what we have reported here is indeed a broadly applicable phenomenon. For example, apart from the previously mentioned styrene polymerisation reaction,^[^ ^18^ ^]^ a similar positive effect on the Grignard reaction was observed in a DES made of ChCl and water,^[^ ^14^ ^]^ as well as in the same DES using other ketones.^[^ ^31^ ^]^ In addition, the propositions of the present work agree with the behaviour of organometallic reactions in flow at room temperature assisted by DES,^[^ ^32^ ^]^ as well as with the reaction of organolithium with nitriles in glycerol.^[^ ^33^ ^]^

Similarly, the *on‐water* chemistry, whereby reactions take place when hydrophobic reagents/catalysts are mixed with water,^[^ ^34^, ^35^ ^]^ or micellar catalysis, where the amphipathic environment shields the organometallic species from the protic solvent,^[^ ^36^ ^]^ may be also thought about using similar concepts.

## Conclusion

Using DES in polar organometallic reactions as the solvent for the substrate can offer drastic improvements in yields and selectivity. In this work, we have analysed the structure of a DES composed of a 1:2 molar ratio of choline chloride and glycerol in presence of an aromatic ketone, acetophenone (AcPh), as a typical substrate for an organolithium or organomagnesium reaction. Wide angle neutron scattering, NMR, tensiometry and neutron reflectivity paired with classical all‐atom molecular dynamics simulations allowed the thorough investigation of the DES (ChCl:Gly) in the presence and absence of AcPh, and the behaviour of the latter in ChCl:Gly and in a (ChCl:Gly)/THF biphase. Structural characterization of ChCl:Gly shows a clear network of H‐bond interactions between choline and glycerol in which chloride anions are accommodated. However, no substantial structural changes within the solvent component interactions were observed in solutions containing the aromatic ketone; the solubility of AcPh in the DES is limited.

Instead, both experiments and calculations agree that there is a tendency for AcPh to move away from ChCl:Gly. This occurs in the case of the (ChCl:Gly)/air interface, where a clear reduction of the surface tension indicates AcPh enrichment at the liquid surface, as well as in the presence of an immobilized hydrocarbon layer in contact with DES.

In full agreement with the experimental observations, MD simulations of a (ChCl:Gly)/THF system revealed the tendency of AcPh to drift from ChCl:Gly into the organic liquid. Moreover, MD simulations of the same system containing *i*‐PrMgCl showed that the latter is unevenly distributed in this biphasic system, and tends to accumulate at the interface.

Altogether, our data rule out the existence of strong specific chemical interactions between any component of the substrate with ChCl:Gly that could enhance its reactivity with respect to the bulk THF environment. Instead, the presence of polar choline chloride in glycerol makes this mixture a less favourable environment for solubilisation of AcPh. Accumulation of AcPh at the ChCl:Gly surface is paired with the tendency of the Grignard compound to locate at the interface between the DES and THF liquids. Thus, rapid stirring while the ethereal organometallic solution is added to the DES/substrate mixture, significantly increasing the contact area, promotes the occurrence of the Grignard reaction at the interface. The stirring also protects the organometallic from a slow decomposition by the ChCl:Gly protons, as it increases the proportion of organic species (in particular the substrate) in the vicinity of the organometallic compound, and explains why the reaction yields drop significantly when the stirring stops.

The selectivity and improved yields promoted by this setup need not be restricted to DES‐based reaction systems but could also be used to engineer reactions using other solvent pairs, for example, by adjusting the relative solubility of the substrate to favour interfacial accumulation. We note that other work has demonstrated the use of aqueous solutions for an *on‐water*‐based reaction system.^[^ ^7^ ^]^ We forecast that studies of the air–solvent surface tension, in the presence of the required substrate, could provide a quick and simple method to screen water, DES or other solvents for their ability to promote similar interfacial reactions arising due to limited solubility of those particular substrates.

## Conflict of Interests

The authors declare no conflict of interest.

## Supporting information

Supporting Information

## Data Availability

Data from the neutron scattering experiments are openly available from the ISIS Neutron and Muon Source website. The liquid diffraction data is at https://doi.org/10.5286/ISIS.E.RB1820315, while the neutron reflectivity data is at https://doi.org/10.5286/ISIS.E.RB2010710‐2. Other data is available in the supplementary information.
