# Supplementary material for: Are Grignard Reactions in Deep Eutectic Solvents Interface‐Driven?
Source: Angew Chem Int Ed Engl. 2025 Sep 1;64(42):e202513649. doi: 10.1002/anie.202513649 (PMC12518705; doi:10.1002/anie.202513649)
Supplement: Supplementary file 1 — Supporting Information [file ANIE-64-e202513649-s001.pdf]

Supporting Information

Are Grignard Reactions in Deep Eutectic Solvents Interface-Driven?

Iva Manasi,+,[a,b] Marco Bortoli,+,[c] Daniel T. Bowron,[d] Mario Campana,[d] Oliver S. Hammond,[e] Thomas F. Headen,[d] Jake Hooton,[b] Eva Hevia,[f] Michele Cascella,*[c] Odile Eisenstein,[c,g] Karen J. Edler*[h]

[a] Department of Physics, University of Bristol, Tyndall Avenue, Bristol, BS8 1TL, United Kingdom

[b] Department of Chemistry, University of Bath, Claverton Down, Bath, BA2 7AX, United Kingdom

[c] Department of Chemistry and Hylleraas Centre for Quantum Molecular Sciences, University of Oslo, PO Box 1033 Blindern, 0315 Oslo, Norway

[d] ISIS Neutron and Muon Source, Rutherford Appleton Laboratory, Oxford, OX11 0QX, United Kingdom

[e] European Spallation Source ERIC, Kongens Lyngby 2800, Denmark

[f] Department für Chemie, Biochemie und Pharmazie, Universität Bern, Freiestrasse 3, 3012 Bern, Switzerland

[g] ICGM, Université de Montpellier, CNRS, ENSCM, 1919 Route de Mende, 34293 Montpellier, France

[h] Department of Chemistry Centre for Analysis and Synthesis (CAS) Lund University, Lund, 221 00 Sweden

[+] These authors contributed equally.
Email: michele.cascella@kjemi.uio.no, karen.edler@chem.lu.se

Contents

S1.Experimental 1

S2.NMR Measurements 2

S3.Liquid Neutron Diffraction Experiments 5

S4.Neutron Reflectivity 12

S5.Molecular Dynamics Simulations 17

References 19

S1. Experimental

Materials

Choline chloride ($[(CH_3)_3NCH_2CH_2OH]Cl$; ChCl; ≥99% chemical purity), glycerol ($HOCH_2CH(OH)CH_2OH$; Gly; ≥99% chemical purity), acetophenone ($CH_3COC_6H_5$; AcPh; 99% chemical purity) and octadecyltrichlorosilane ($CH_3(CH_2)_17SiCl_3$; OTS; ≥90% purity) were obtained from Sigma-Aldrich. d9-choline chloride ($[(CD_3)_3NCH_2CH_2OH]Cl$; d-ChCl; 98 atom-% D), d8-glycerol ($DOCD_2CD(OD)CD_2OD$; d-Gly; 99 atom-% D) and d5-acetophenone ($CH_3COC_6D_5$; d-AcPh; 98 atom-% D) were purchased from Cambridge Isotope Laboratories. All chemicals were used as provided. The silicon blocks used for neutron reflectivity were sourced from Crystran Limited.

Sample Preparation

The choline chloride:glycerol (ChCl:Gly) DES was prepared by combining the components in molar ratios of 1:2. The mixtures were stirred at 60 °C until a clear, homogeneous liquid was obtained, which was subsequently sealed and equilibrated overnight. Acetophenone was dissolved in the DES or glycerol by vigorous magnetic stirring at room temperature at different concentrations (0.0008 – 1 mmol g-1) for tensiometry experiments or different isotopically labelled contrasts in saturated solutions for liquid neutron diffraction and reflectometry measurements.

Experimental measurements

To quantify the solubility of acetophenone in ChCl:Gly DES and in glycerol, 1H NMR spectra were acquired at 298 K on a Bruker Neo500 spectrometer operating at 500.13 MHz, using 4 scans, 30-degree pulses, and a 60 second delay between pulses to help ensure accurate quantitation.

Liquid neutron diffraction experiments were performed using the NIMROD diffractometer [1] at ISIS Pulsed Neutron and Muon Source, UK (RB1820315 [2]). Using time-of-flight (TOF) neutrons with wavelength $0.05 \leq \lambda \leq 11$ Å and detectors spanning the angular range 0.5 - 40°, a Q-range of $0.01 \leq Q \leq 50$ Å^{-1} was obtained. Pure DES samples were prepared and measured at 6 different isotopically labelled contrasts for liquid neutron diffraction experiments. Acetophenone in DES mixtures were prepared at 1 mmol g^{-1} and measured at the saturated concentration at 8 isotopically labelled contrasts. In case of acetophenone in glycerol 1 mmol g^{-1} mixtures were prepared and measured at the saturated concentration at four isotopically labelled contrasts. See Section S3 for details.

Null-scattering, vacuum-sealed $Ti_{0.68}Zr_{0.32}$ sample cells with a path-length of 1 mm were filled with 1.5 g of each sample and placed in a sample changer at 298 ± 0.1 K. Measurements were performed using a collimated neutron beam of 30 mm square, with a counting time of 2˘3

hours. Empty sample cells, the empty instrument, and a 3 mm thick null coherent scattering vanadium niobium standard were measured for data normalisation and instrument calibration. Sample scattering patterns were processed using GudrunN software [3] by correcting for attenuation, multiple scattering and the sample environment background, and normalising the data using the vanadium standard measurement.

After correction for hydrogen inelasticity, [4] the reduced data were analysed using Empirical Potential Structure Refinement (EPSR) modelling, [5–7] a Monte Carlo-derived method, that uses the experimental diffraction data to constrain a molecular model.

Surface tension measurements were made at room temperature (21 °C) using the pendant-drop-shape-analysis method [8] with a FTA1000 Drop Shape Analyser. Concentration series of the acetophenone in DES and acetophenone in glycerol ranging from $0.0008 - 1$ mmol g^{-1} ($0.01 - 12$ wt%) were prepared and measured at the air interface. The surface tension at each concentration was measured $3 - 4$ times using a fresh drop, with each measurement comprising 100 data points taken within 1 min. The data reported is the average value of the measurements with the standard error.

Neutron reflectivity was measured on the OFFSPEC reflectometer [9] at ISIS Pulsed Neutron and Muon Source, UK (experiment number RB2010710 [10]). Using time-of-flight (TOF) neutrons with wavelengths $1.0 \leq \lambda \leq 14$ Å and incidence angles of $0.7°$ and $2.0°$, a Q-range of $0.012 \leq Q \leq 0.27$ Å^{-1} was obtained.

Silicon blocks for reflectivity were coated with an OTS layer, which mimics the hydrophobic interface that is present in the reaction more closely than air, using the procedure described by Brzoska et al. [11] The OTS coated silicon blocks were sealed into a solid-liquid reflectivity flow cells through which the ChCl:Gly DES was flowed, without and with the acetophenone at three contrasts of the solvent/acetophenone. The multiple neutron reflectivity contrasts were co-refined to a layered structure using the RasCAL fitting routine, [12] allowing for determination of the presence and penetration of acetophenone at the solvent/OTS boundary and giving molecular details of the interfacial layer. Confidence bands on the fit parameters and the structure were determined using RasCAL's inbuilt Bayesian analysis function. Details for the method, model and fitting are given in Section S4.

S2. NMR Measurements

NMR measurements were done on solutions recovered after wide angle neutron scattering experiments, to quantify the solubility of acetophenone in ChCl:Gly DES and in glycerol. Figure S1 shows the NMR spectra obtained from ChCl:Gly DES and ChCl:Gly DES with 1 mmol/g of added AcPh. The relative ratio of the CH_3 peak integral for choline (position 3.19 ppm and integral, $I_{Ch} = 9$) and AcPh (position 2.58 ppm and integral, $I_{AcPh} = 0.19$) was used to calculate the molar ratio of AcPh to choline as $\frac{I_{AcPh}/3}{I_{Ch}/9} = 0.063$. Converting this to AcPh concentration in the DES using the choline concentration in the DES, this gives us 0.195 mmol g^{-1} of AcPh in

the DES, indicating that only a fifth of the AcPh added to the solution is dissolved in the DES.

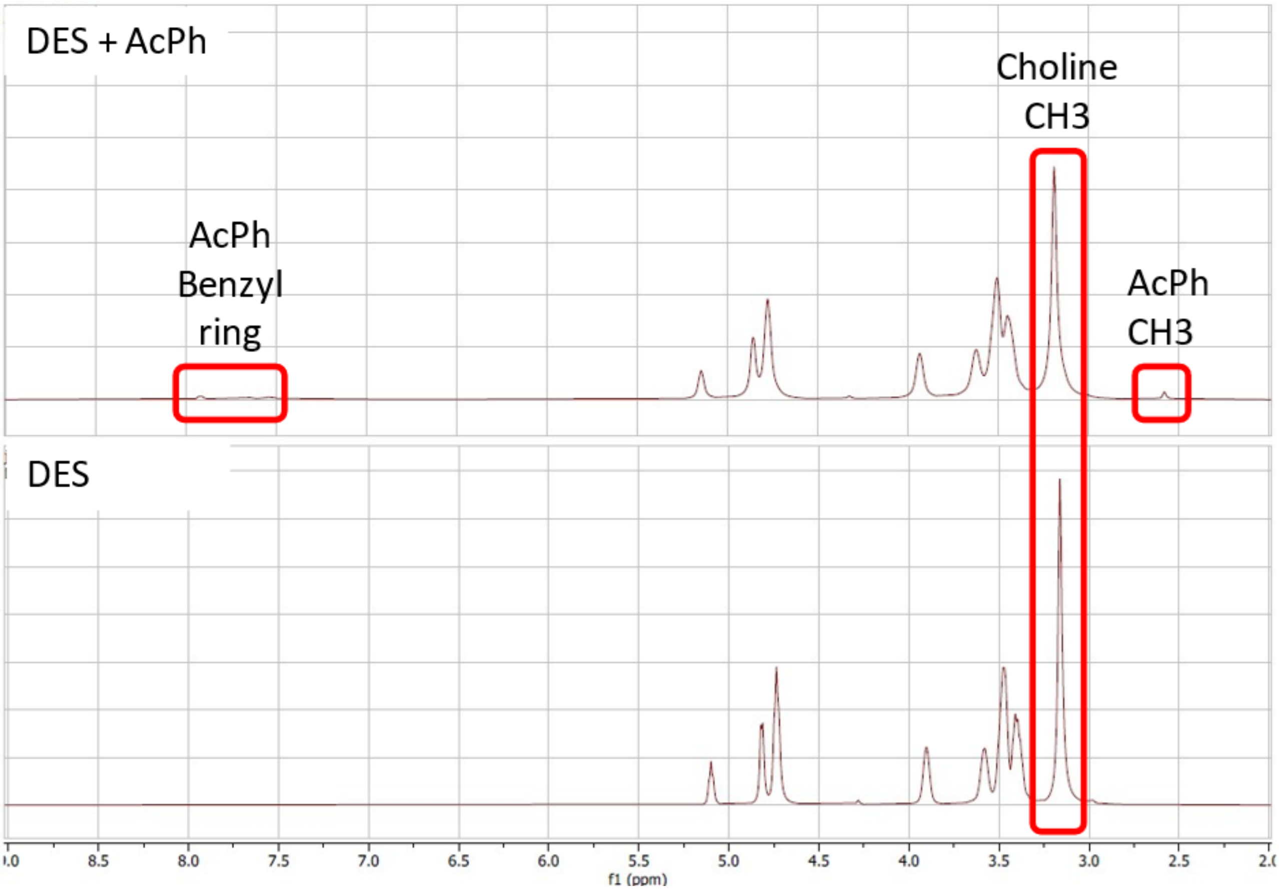

Figure S1 NMR spectra from ChCl:Gly DES (bottom) & 1 mmol g^{-1} of AcPh in ChCl:Gly DES (top).

A similar procedure was done for acetophenone in glycerol, shown in Figure S2. Here we use the relative ratio of the 2*CH2 and 1*CH peaks integrals in glycerol (peak position and integral $I_{Gly} = 5$) to the CH_3 peak integral in AcPh (peak position and integral $I_{AcPh} = 0.0419$) to calculate the molar ratio of AcPh to glycerol as $\frac{I_{AcPh}/3}{I_{Gly}/5} = 0.014$. This gives 0.15 mmol g^{-1} of AcPh in glycerol for an added concentration of 0.2 mmol g^{-1} and 0.76 mmol g^{-1} AcPh in glycerol for an added concentration of 1 mmol g^{-1}, indicating three quarters of the AcPh added to the solution is dissolved in the glycerol.

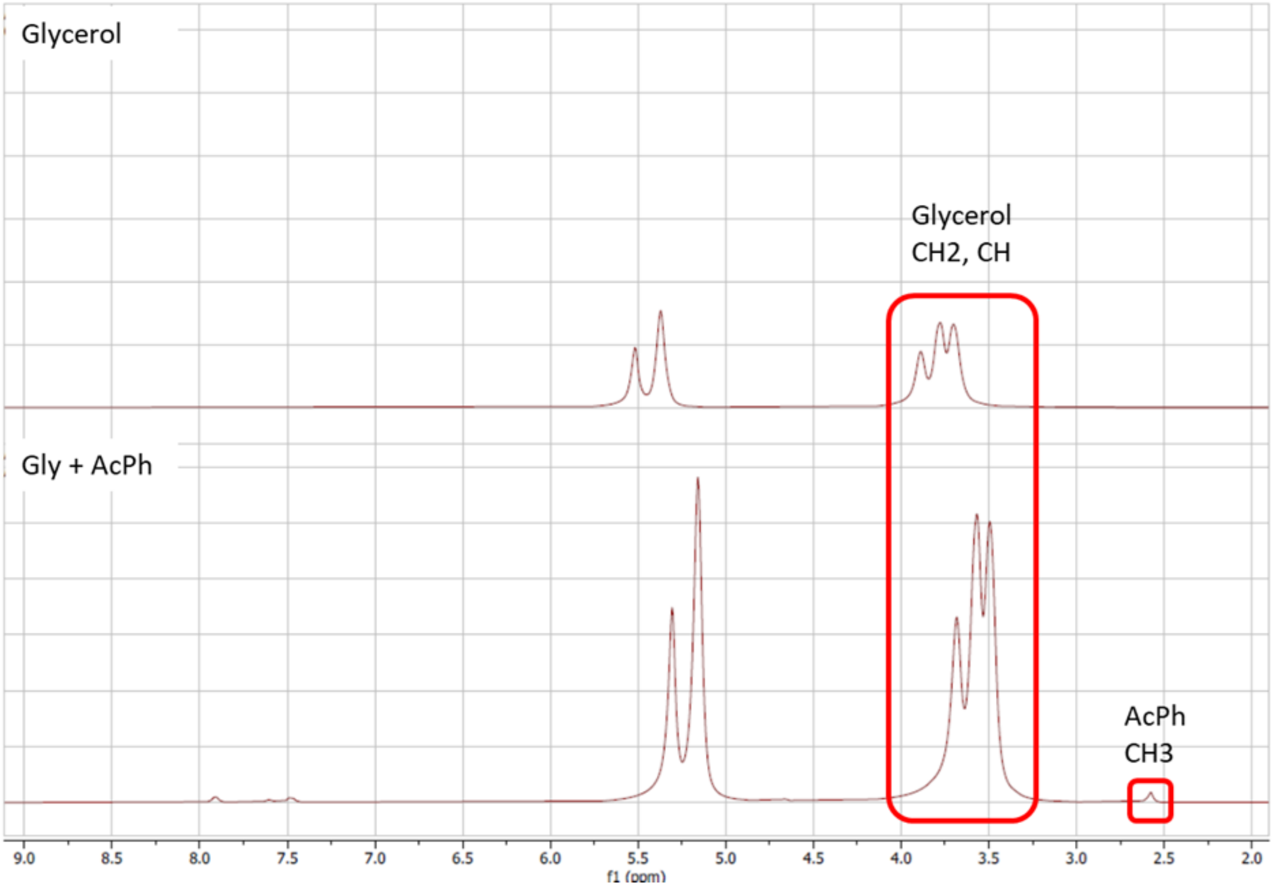

Figure S2 NMR spectra from Glycerol DES (top) & 0.2 mmol g^{-1} of AcPh in Glycerol DES (bottom).

S3. Liquid Neutron Diffraction Experiments

Pure DES samples were prepared and measured at 6 different isotopically labelled contrasts for liquid neutron diffraction experiments; h-ChCl:h-Gly (H:H), h-ChCl:d-Gly (H:D), d-ChCl:h-Gly (D:H), d-ChCl:d-Gly (D:D), equimolar h- & d-ChCl:d-Gly (H/D:D) and d-ChCl:equimolar h- & d-Gly (D:H/D). Acetophenone in DES mixtures were prepared and measured at 8 isotopically labelled contrasts at a desired concentration of 1 mmol/g for the h-AcPh in h-ChCl:h-Gly contrast and equivalent molar ratios for other isotopically labelled samples; h-ChCl:h-Gly:h-AcPh (H:H:H), h-ChCl:d-Gly:d-AcPh (H:D:D), d-ChCl:h-Gly:d-AcPh (D:H:D), d-ChCl:d-Gly:h-AcPh (D:D:H), d-ChCl:d-Gly:d-AcPh (D:D:D), equimolar h- & d-ChCl:equimolar h- & d-Gly:equimolar h- & d-AcPh (H/D:H/D:H/D), d-ChCl:d-Gly:equimolar h- & d-AcPh (D:D:H/D) and d-ChCl: equimolar h- & d-Gly:d-AcPh (D:H/D:D). In case of Acetophenone in glycerol samples were prepared and measured at 4 isotopically labelled contrasts at a desired concentration of 1 mmol/g for the h-AcPh in h-Glycerol contrast and equivalent molar ratios for the other isotopically labelled samples; h-Gly:d-AcPh (H:D), d-Gly:h-AcPh (D:H), d-Gly:d-AcPh (D:D), equimolar h- & d-Gly:d-AcPh (H/D:D). We note that the NMR measurements described in S1 above showed that the actual amount of acetophenone in the DES mixtures was 0.2 mmol g^{-1} while in glycerol it was 0.75 mmol g^{-1}.

In each case, the accurate mass of each sample was recorded (weighing error $\pm$ 0.1 mg) to determine the sample purity. This was achieved by comparing the measured neutron scattering differential cross section (DCS) with the calculated DCS from the sample masses, using GudrunN neutron total scattering data reduction software.[3] Sample purity was determined to be adequate, as assessed by being within a neutron DCS error margin of $\pm$5% for fully deuterated samples, and $\pm$10% for fully hydrogenous samples. This is within the error of the diffraction experiment and sample preparation, with a higher error for more proton-rich samples because of the strong inelastic scattering of neutrons by hydrogen nuclei.[4] After a final correction for hydrogen inelasticity, the reduced data were analysed using Empirical Potential Structure Refinement (EPSR) modelling.[5]

EPSR simulates a 3D configuration that is objectively consistent with experimentally determined diffraction data for a system. This is achieved by using standard Lennard-Jones parameters and the known quantities of the system, such as molecular geometry and structure, density, and composition, as well as permitting intramolecular disorder. For the ChCl:Gly DES, 200 choline, 200 chloride and 400 glycerol molecules were added to a simulation box of 45 Å^3 with an atomic density of 0.11 atom Å^{-3}. In case of ChCl:Gly DES with acetophenone, 200 choline, 200 chloride, 400 glycerol and 14 acetophenone molecules in a box of 45.3 Å^3 with the same atomic density were used, and in the case of glycerol with acetophenone, 400 glycerol and 37 acetophenone molecules were taken in a box of 45.3 Å^3 with an atomic density of 0.112 atom Å^{-3}. The required number of acetophenone molecules was calculated from the concentration in each system, as determined by NMR. The labelling of molecules used in the

simulation are shown in Figure S3, alongside their parameterization, in Table S1.

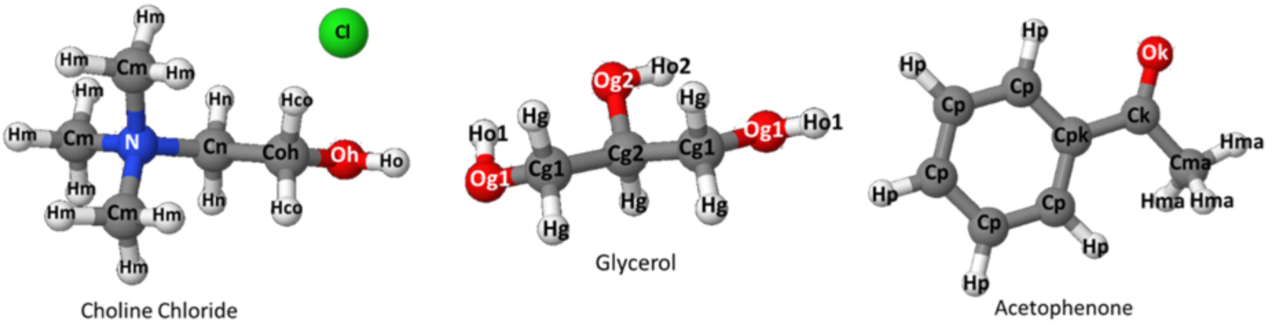

Figure S3 Labelling of atoms in the various molecules (choline chloride, glycerol and acetophenone) used in EPSR modelling.

Table S1 Lennard-Jones parameters, including the charges and masses for the different atoms of choline chloride, glycerol and acetophenone used in the reference potential for EPSR modelling.

Atom Type	ε/kJ mol^{-1}	σ/Å	mass/amu	q/e
**Choline Chloride**				
**N**	0.7	3.2	14	1
**Cn**	0.8	3.7	12	-0.12
**Cm**	0.8	3.7	12	-0.18
**Hn**	0.2	2.58	2	0.06
**Hm**	0.2	2.58	2	0.06
**Coh**	0.8	3.7	12	0.145
**Hco**	0.2	2.58	2	0.06
**Oh**	0.65	3.1	16	-0.683
**Ho**	0	0	2	0.418
**Cl-**	0.566	4.191	35.5	-1
**Glycerol**				
**Cg1**	0.8	3.7	12	0.107
**Cg2**	0.8	3.7	12	0.17
**Hg**	0.2	2.58	2	0.062
**Og1**	0.65	3.1	16	-0.624
**Og2**	0.65	3.1	16	-0.624
**Ho1**	0	0	2	0.392
**Ho2**	0	0	2	0.392
**Acetophenone**				
**Cpk**	0.273	3.7	12	0.15
**Cp**	0.293	3.7	12	-0.115
**Ck**	0.441	3.7	12	0.32
**Hp**	0.126	2.42	2	0.115
**Ok**	0.882	2.96	16	-0.47
**Cma**	0.273	3.5	12	-0.18
**Hma**	0.2	2.58	2	0.06

The model was allowed to run for a number of MC cycles until it equilibrated in energy, whilst being compressed by approximately 10% per cycle until stabilizing at the experimentally measured density.[13] The empirical potential was then introduced to refine the model to the neutron diffraction data, and approximately 10000 refinement cycles were performed to accumulate statistics on the structural information including intermolecular coordination numbers, radial distribution functions (RDFs), the spatial density functions (SDFs) and the intermolecular coordination numbers between different components of the system using the COORD routine of EPSR. The molecular centres are defined as the N atom for choline, Cg2 atom for glycerol, Cpk atom for acetophenone and for the monoatomic Cl^- it is the true centre of mass.

The experimental datasets along with the fits from Empirical Potential Structure Refinement atomistic models for the ChCl:Gly DES are shown in Figure S4, for the ChCl:Gly DES with 1 mmol g^{-1} of acetophenone (actual dissolved concentration of 0.2 mmol g^{-1}) in Figure S5 and glycerol with 1 mmol g^{-1} of acetophenone (actual dissolved concentration of 0.75 mmol g^{-1}) in Figure S6. Inspection of the experimental diffraction patterns with EPSR fits reveals that the EPSR model has been able to equilibrate to the experimental data very closely and transforming the data into r-space further demonstrates the quality of this fit. EPSR calculates a value indicating the quality of fit called the R-factor, where a low R-factor means a better fit. The mean R-factor value over the approximately 10000 iterations accumulated over the refinement procedure was < 0.01, a comparatively low value indicating that the EPSR model is as objectively representative of the experimental data as it is possible for it to be.[6] It is noted that the major source of discrepancy occurs at $Q \leq 2$ Å^{-1}, which is the region affected by the inelastic scattering of light hydrogen. It was not possible to obtain perdeuterated choline chloride, and so the disagreement here between model and experimental data for all isotopic contrasts can be attributed to slight over- or under-subtraction of the substantial wavevector-dependent inelastic scattering background that light hydrogen produces. This is a known issue in the analysis of data from neutron scattering experiments.[4,14] The correlation peak and the first minima for the principle RDFs of the ChCl:Gly DES are given in Table S2 along with the values obtained for the DES at 60 °C by Turner and Holbrey[15] and the values for ChCl:Gly DES and glycerol with 1 mmol g^{-1} of acetophenone are given in Table S3.

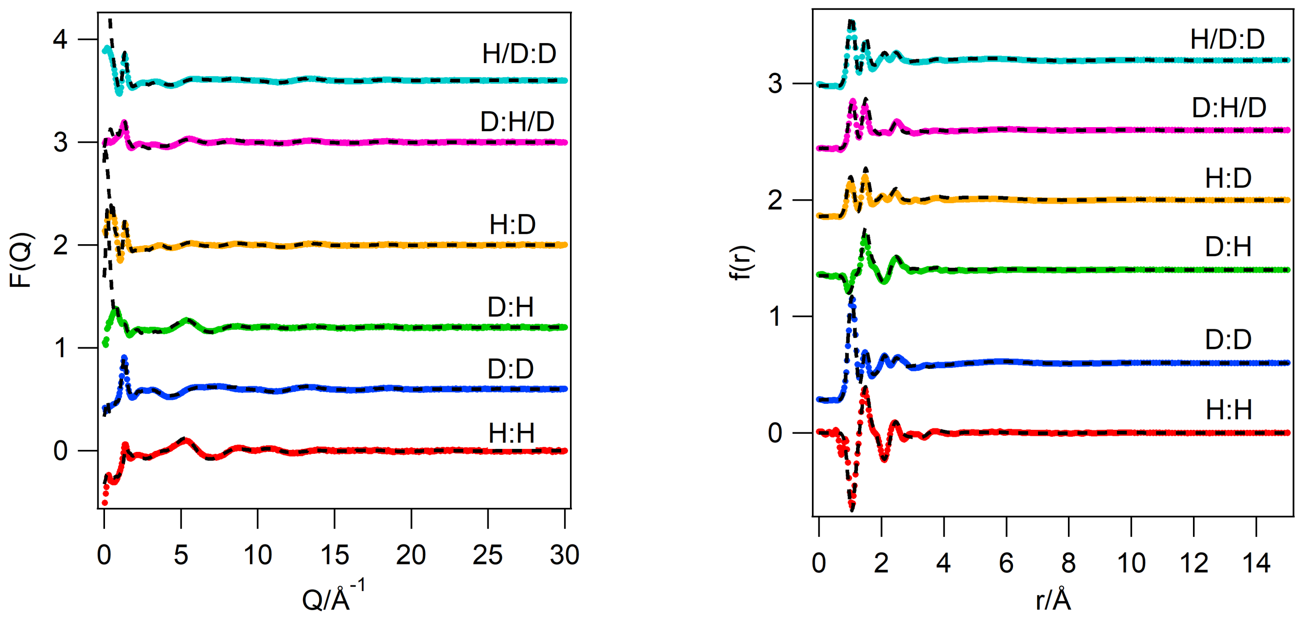

(a) (b)

Figure S4 EPSR fits (dashed black lines) to the ChCl:Gly DES liquid neutron diffraction data (coloured circles). (a) F(Q) vs Q, diffraction data in reciprocal space. (b) f(r) vs r, Fourier transform of diffraction data in real space. The contrast of the components is indicated in the data labels, in the order Ch:Gly. The data is offset along the y-axis for clarity.

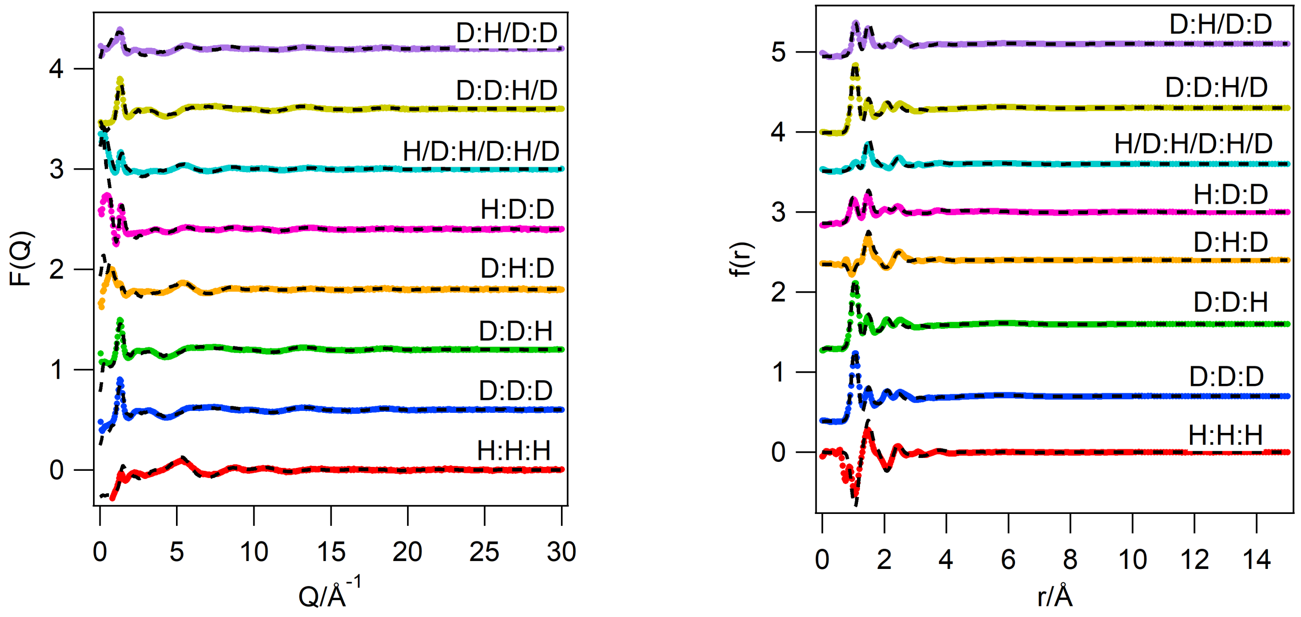

(a) (b)

Figure S5 EPSR fits (dashed black lines) to the ChCl:Gly DES with acetophenone liquid neutron diffraction data (coloured circles). (a) F(Q) vs Q, diffraction data in reciprocal space. (b) f(r) vs r, Fourier transform of diffraction data in real space. The contrast of the components is indicated in the data labels, in the order Ch:Gly:AcPh. The data is offset along the y-axis for clarity.

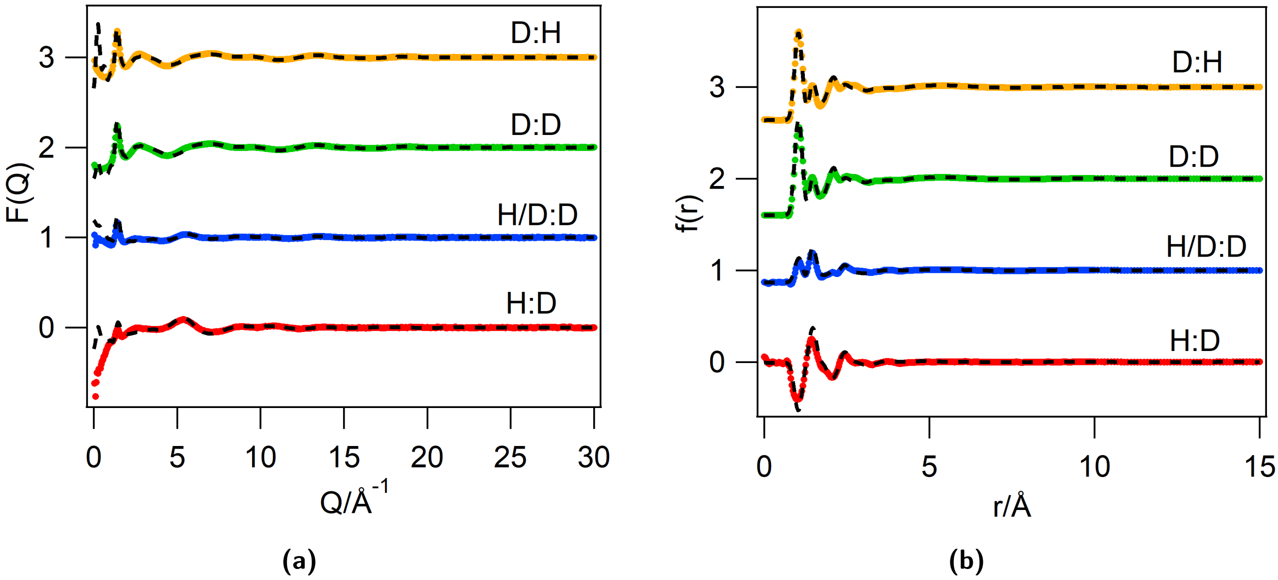

Figure S6 EPSR fits (dashed black lines) to glycerol with acetophenone liquid neutron diffraction data (coloured circles). (a) F(Q) vs Q, diffraction data in reciprocal space. (b) f(r) vs r, Fourier transform of diffraction data in real space. The contrast of the components is indicated in the data labels, in the order Gly:AcPh. The data is offset along the y-axis for clarity.

Table S2 Primary correlation peak (r_{max}) and first minima (r_{min}) for the principle RDFs (pRDFs) of the ChCl:Gly DES. The values as observed by Turner & Holbrey[15] at 60 °C are given for comparison.

pRDF	ChCl:Gly Turner & Holbrey; 60 °C $r_{max}(r_{min})$/Å	ChCl:Gly this work; 25 °C $r_{max}(r_{min})$/Å	pRDF	ChCl:Gly Turner & Holbrey; 60 °C $r_{max}(r_{min})$/Å	ChCl:Gly this work; 25 °C $r_{max}(r_{min})$/Å
**Choline-Choline**			**Glycerol-Glycerol**		
N-N	5.5 (6.4)	6.2 (6.8)	Cg2-Cg2	5.2 (7.5)	5.3 (7.3)
Cn-Cn	6.4 (8.1)	6.5 (8.6)	Cg2-Cg1	4.9 (7.5)	5.2 (7.6)
Cn-N	6.2 (7.2)	6.4 (7.7)	Cg2-Og2	4.9 (5.4)	4.8 (5.3)
N-Coh	4.6 (5.6)	5.3 (6.4)	Cg2-Og1	4.9 (7.2)	4.8 (6.9)
N-Oh	4.9 (6.8)	4.6 (6.4)	Cg1-Cg1	5.7 (7.4)	5.9 (6.9)
N-Ho	5.2 (7.2)	4.7 (6.9)	Cg1-Og1	4.8 (7.3)	5.0 (6.2)
Oh-Ho	3.5 (4.5)	3.4 (3.9)	Og2-Og2	2.7 (3.2)	2.8 (3.4)
**Choline-Chloride**			Og2-Og1	2.7 (3.1)	2.7 (3.5)
N-Cl	4.3 (6.5)	4.5 (7.1)	Og2-Hg1	1.8 (2.4)	1.8 (2.4)
Cn-Cl	3.6 (5.2)	3.9 (5.2)	Og1-Og1	2.7 (3.1)	2.7 (3.6)
Cl-Hm	2.8 (3.4)	3.0 (4.0)	Og1-Hg1	1.7 (2.3)	1.7 (2.4)
Cl-Hn	2.8 (4.2)	2.0 (4.1)	Hg1-Hg1	2.4 (3.4)	2.4 (3.4)
Cl-Hco	3.3 (4.3)	3.1 (4.3)	**Glycerol-Chloride**		
Cl-Ho	2.2 (3.5)	2.1 (3.1)	Cl-Cg2	3.9 (4.7)	3.9 (4.6)
**Choline-Glycerol**			Cl-Hg1	2.1 (3.4)	2.1 (3.3)
N-Cg2	5.4 (7.8)	5.5 (7.6)	**Chloride-Chloride**		
Cn-Cg2	6.4 (8.3)	5.8 (8.0)	Cl-Cl	7.1 (10.3)	
Hm-Og1	2.9 (3.4)	2.6 (3.2)			
Hn-Og1	3.4 (4.2)	2.7 (3.5)			
Hco-Og1	3.6 (4.1)	2.7 (3.5)			
Oh-Hg1	3.4 (4.3)	3.3 (4.0)			
Ho-Og1	1.9 (2.5)	1.8 (2.5)			

Table S3 Primary correlation peak (r_{max}) and first minima (r_{min}) for the principle RDFs (pRDFs) of the acetophenone centres in ChCl:Gly DES and glycerol with acetophenone.

	**AcPh in ChCl:Gly**	**AcPh in Glycerol**
**pRDF**	$\mathbf{r_{max}(r_{min})}$/Å	$\mathbf{r_{max}(r_{min})}$/Å
**AcPh-AcPh**		
Ok-Ok	7.5 (8.1)	7.2 (8.6)
**AcPh-Glycerol**		
Ok-Og1	2.7 (3.2)	2.7 (3.3)
Ok-Og2	2.7 (3.2)	2.7 (3.4)
Ok-Ho1	1.8 (2.7)	1.8 (2.7)
Ok-Ho2	1.9 (2.6)	1.8 (2.8)
**AcPh-Choline**		
Ok-N	4.5 (6.2)	
Ok-Ooh	2.7 (3.3)	
Ok-Hoh	1.7 (2.7)	
**AcPh-Chloride**		
Ok-Cl	3.1 (4.3)	

S4. Neutron Reflectivity

Neutron Reflectivity was measured on a model system consisting of a hydrophobic OTS layer supported on silicon blocks, exposed to the solutions of ChCl:Gly. The silicon blocks for the neutron reflectivity measurements were coated with an OTS layer, which mimics the hydrophobic interface that is present in the reaction more closely than air, using the procedure described in Brzoska et al.[11] The silicon blocks were cleaned using a piranha solution (5:1:1 mixture of water, conc sulfuric acid and 30 wt% hydrogen peroxide) followed by a basic RCA cleaning solutions (5:1:1 mixture of water, 29 wt% ammonia water and 30 wt% hydrogen peroxide). The silicon blocks were then sonicated in methanol for 5 mins, followed by 1:1 mixture of methanol and chloroform for 5 mins and finally chloroform for 5 mins. They were left until the chloroform evaporated and then blow-dried using nitrogen. Once cleaned the silicon blocks were placed in a large petri dish containing chloroform inside a glove bag filled with nitrogen. Octadecyltrichlorosilane (OTS) is then injected into the petri dish as close to the silicon blocks as possible (1 vol% of the chloroform). The petri dish was then covered, and the silicon blocks allowed to sit in the silane chloroform mixture for 2 hours. Once the process is complete, the blocks were taken out of the glove bag and a reverse solvent clean was done in the sonicator; chloroform for 5 mins, followed by 1:1 chloroform/methanol mixture followed by methanol. Finally, the blocks were blow-dried using nitrogen and the silicon blocks should be coated with a monolayer of the OTS, testing by checking the wettability of water on the surface (Figure S7).

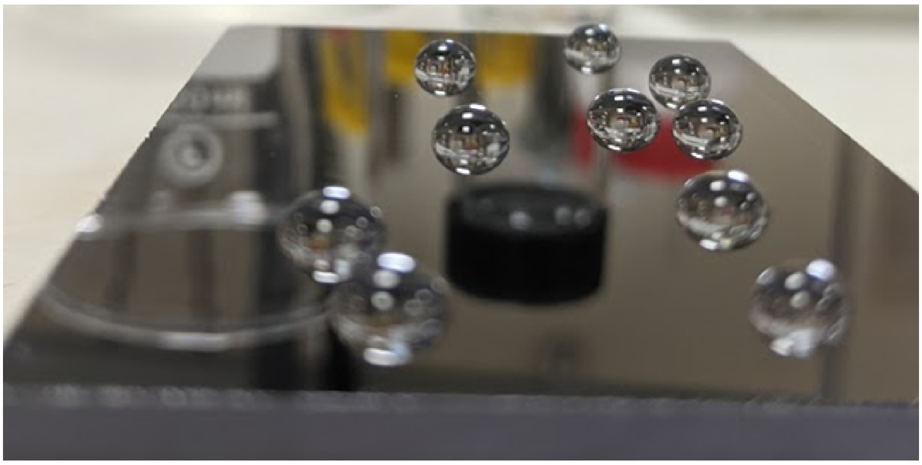

Figure S7 Silicon block with water droplets showing hydrophobization of the block confirming coating with an octadecyltrichlorosilane (OTS) layer.

The OTS coated silicon blocks were used in sealed solid-liquid reflectivity flow cells through which the ChCl:Gly DES was flowed, without and with the AcPh additive. Informed by the surface tension data, which suggests an interface saturated with acetophenone at that concentration, a concentration of 0.2 mmol g^{-1} was chosen for the neutron reflectivity measurements to study the molecular details of the interfacial acetophenone layer. Neutron reflectivity was measured from with the neutrons incident through the silicon block making a grazing angle

of incidence with the OTS/DES interface from the silicon super-phase. This presents a layered structure of silicon oxide capped silicon followed by the OTS layer and the DES (or AcPh interfacial layer and then DES). The layered structure and the neutron scattering length densities (SLD) of various components in the system as summarised in Table S4.

Measurements were done at three contrasts of ChCl:Gly DES and AcPh: d-AcPh in h-ChCl:h-Gly (h-DES), h-AcPh in d-ChCl:d-Gly (d-DES) and d-AcPh in d-ChCl:d-Gly (d-DES), as well as the OTS/air interface for both the silicon substrates used in the study. There is very little change in the reflectivity profile without and with d-AcPh for d-DES. This could be due to an insufficient contrast between the d-AcPh (especially when solvated) and d-DES. Therefore, this dataset was omitted from the co-refining routine. For d-AcPh in h-DES and h-AcPh and d-DES, the layered structure with the SLDs of the various components were used to co-refine the two neutron reflectivity contrasts using the RasCAL fitting routine.[12] Each layer (silicon oxide and interface) in the model is defined by the SLD, thickness and interfacial width. The silicon dioxide layer is parameterized by the SLD (as given in Table S4), a thickness, a Nevot-Croce roughness.[16] The interfacial region is divided into two layers: Layer 1 and Layer 2. Both layers are parametrized by a thickness, Nevot-Croce roughness and volume fraction of OTS (ϕ_{OTS}); higher in Layer 1 and smaller in Layer 2[††]). When the measurement is done with AcPh in the DES, the AcPh can insert into the two layers, parameterized by the volume fraction of AcPh (ϕ_{AcPh}) in each of the layers. The rest of the volume in the layers is taken up by the DES ($\phi_{DES} = 1 - \phi_{OTS} - \phi_{AcPh}$), i.e. the solvation of each layer. The SLD of the each of the two layers is calculated by multiplying the volume fraction of the component with the SLD at that contrast (given in Table S4) which is then fed into the model.

The silicon oxide layer thickness and roughness, and the OTS layer thickness, roughness and volume fraction were constrained between the 6 measured reflectivity curves (OTS/air for the two silicon substrates,[‡‡] OTS/h-DES, OTS/d-DES, OTS/h-AcPh+d-DES and OTS/d-AcPh+h-DES), the AcPh layer thickness, roughness and volume fraction were constrained between the 2 measured reflectivity curves (OTS/h-AcPh+d-DES and OTS/d-AcPh+h-DES and the data was co-refined to fit the 10 parameters: silicon dioxide thickness and roughness, layer 1 thickness, roughness, ϕ_{OTS} and ϕ_{AcPh}, and layer 2 thickness, roughness, ϕ_{OTS} and ϕ_{AcPh}. Confidence bands on the fit parameters and the structure were determined using RasCAL's inbuilt Bayesian analysis function. The neutron reflectivity data along with the fits and the corresponding SLD profile along with the 65% confidence interval on the parameters is shown in Figure S8 and Figure 5 in the main paper. The SLD distribution was used to get the volume fraction distribution of all components (silicon, silicon dioxide, OTS, DES and AcPh) across the interface, as

[††]Layer 2 can comprise a small amount of OTS as there may be small aggregates that may have been deposited during the silanization process.

[‡‡]Given the cleaning and silanization procedure is the same between the different substrates, the difference between the silicon oxide layer and OTS layer should be within measurement resolution. Therefore, to restrict the number of fit parameters, these were constrained between the two silicon substrates and measured contrasts (h-AcPh in d-DES and d-AcPh in h-DES).

Table S4 Scattering Length Density of the components used to refine the neutron reflectivity layers. The schematic to the right shows the experimental set up, with the silicon substrate at the base of the cell (native oxide layer not shown), the OTS layer and the DES solution on top, with an interfacial acetophenone layer when acetophenone is added to the solution.

	Scattering Length Density (SLD) / 10^{-6} Å^{-2}	
	**Protonated contrast**	**Deuterated contrast**
**Silicon**	2.07	NA
**Silicon Oxide**	3.0	NA
**OTS layer**	0.37	NA
**DES**	0.45	6.0
**Acetophenone**	1.88	4.2

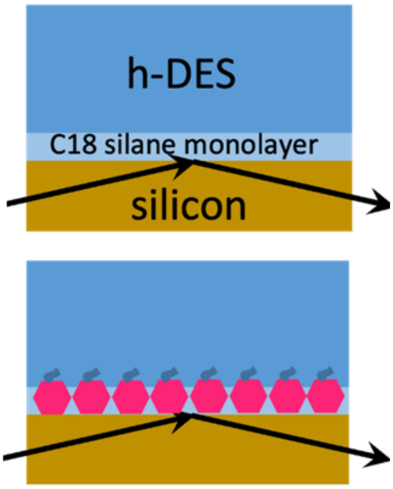

shown in Figure 5 in the main paper. The posterior distribution for the fit parameter for Layer 1 and Layer 2 assuming uniform priors and their best fit values with 65% and 95% confidence interval are given in Figure S9 and Table S5, respectively. A schematic depicting this layer structure, based on the volume fraction distribution of the components from the reflectivity fits, is shown in Figure S10.

Table S5 Best fit values obtained for the fit parameters of the reflectivity data and their 65% and 95% confidence intervals using Bayesian analysis.

Parameter	Best fit	95% CI	65% CI
**Layer 1 thickness (Å)**	32.5	[23.0, 42.1]	[28.0, 37.5]
**Layer roughness (Å)**	2.6	[2.1, 7.7]	[2.7, 6.1]
**Layer 1 ϕ_{OTS}**	0.205	[0.160, 0.372]	[0.188, 0.313]
**Layer 1 ϕ_{AcPh}**	0.395	[0.207, 0.463]	[0.276, 0.418]
**Layer 2 thickness (Å)**	41.0	[31.9, 72.0]	[37.9, 59.7]
**Layer roughness (Å)**	2.6	[2.1, 7.7]	[2.7, 6.1]
**Layer 2 ϕ_{OTS}**	0.046	[0.008, 0.077]	[0.027, 0.060]
**Layer 2 ϕ_{AcPh}**	0.102	[0.025, 0.167]	[0.050, 0.134]

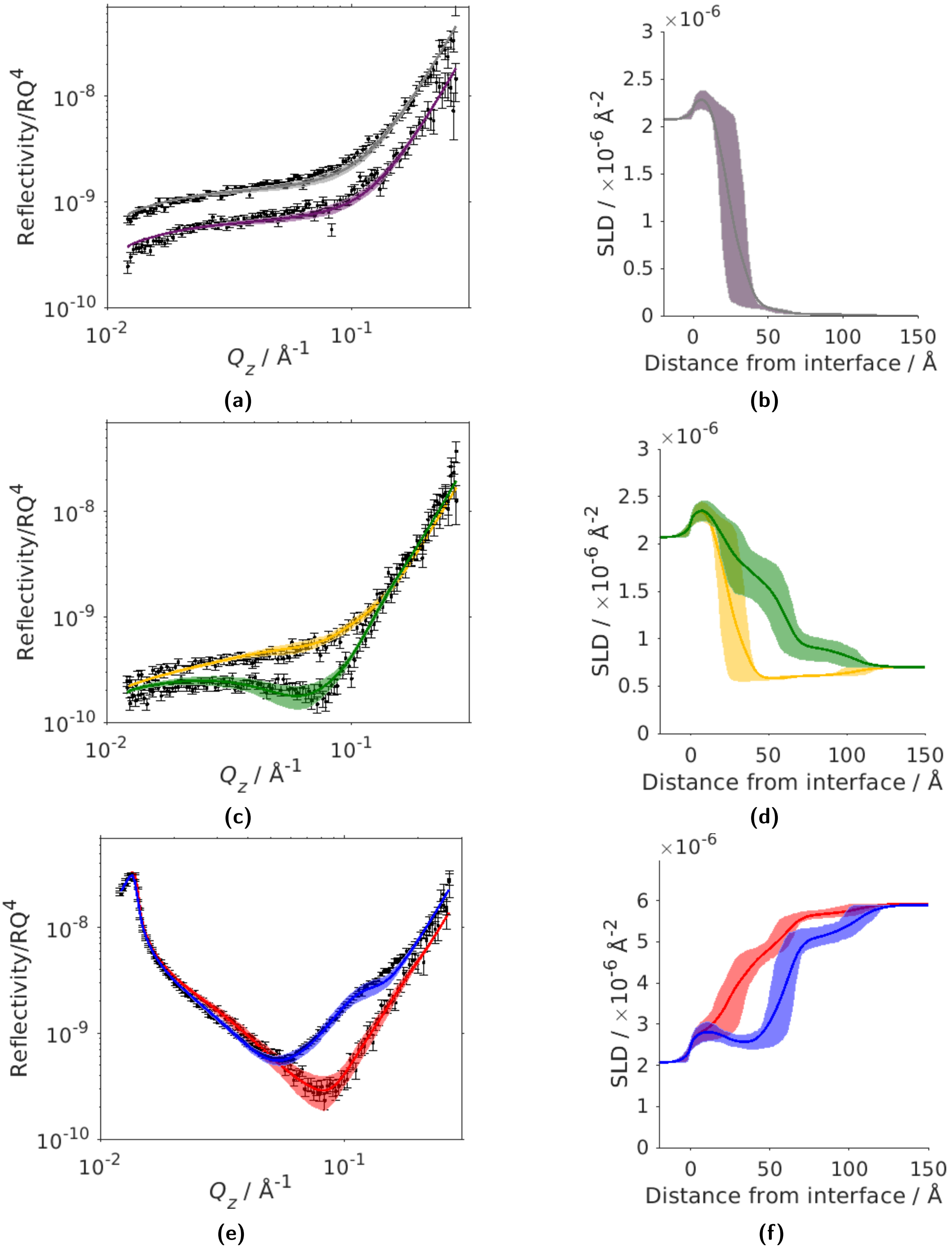

Figure S8 Neutron reflectivity measurements and the fits for ChCl:Gly/OTS interface without and with added acetophenone (left panel) along with the SLD profile of the fitted interfacial layered structure (right panel). (a) & (b): Silicon/OTS/air interface for the two substrates (one for h-DES measurements and other for d-DES measurements). They are virtually identical and have been offset along the y-axis to show the fit quality of both. (c) & (d): Silicon/OTS/h-DES interface before (yellow) and after (green) the introduction of acetophenone. (e) & (f): Silicon/OTS/d-DES interface before (red) and after (blue) the introduction of acetophenone. The shaded areas in the plots represent the 65% confidence interval.

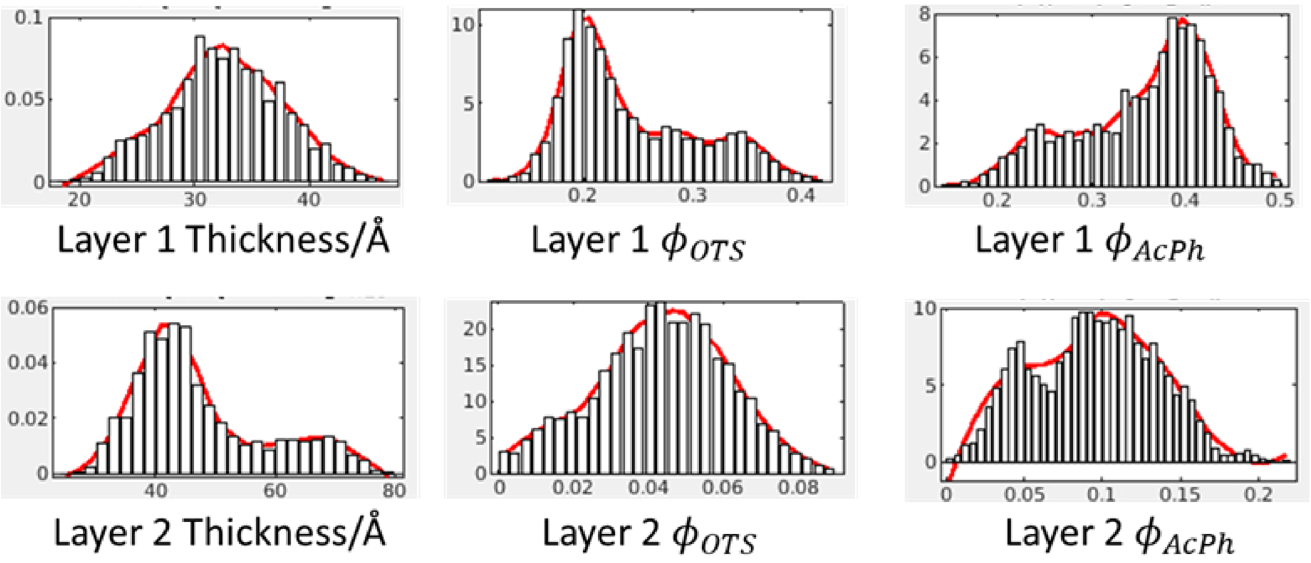

Figure S9 The posterior distribution for the reflectivity fit parameters assuming uniform priors using Bayesian analysis.

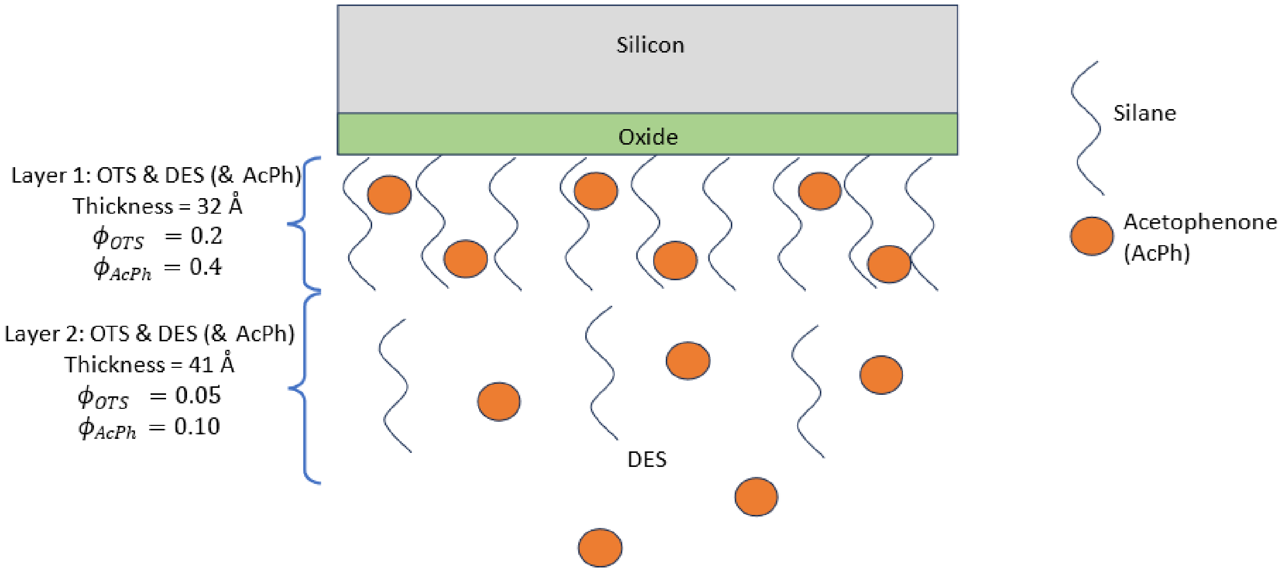

Figure S10 A schematic showing the layered structure used for the modelling of the reflectivity data along with thickness and volume fraction parameters fits from the reflectivity data.

S5. Molecular Dynamics Simulations

Table S6 Average number of H-bonds per acceptor molecule for the pure DES, and a 0.2 mmol g^{-1} solution of AcPh in DES at 300 K obtained from MD simulations.

		Average H-bond number	
Donor	Acceptor	pure DES	AcPh/DES
Ch^+	Cl^-	0.54 ± 0.02	0.54 ± 0.02
Ch^+	Gly (O)	0.10 ± 0.02	0.10 ± 0.01
Ch^+	AcPh		0.03 ± 0.03
Gly	Cl^-	1.89 ± 0.04	1.90 ± 0.05
Gly (HO)	Gly (O)	0.66 ± 0.02	0.65 ± 0.02
Gly (HO)	Ch^+	0.06 ± 0.10	0.05 ± 0.10
Gly	AcPh		0.27 ± 0.09

Table S7 Diffusion coefficient for a solution of AcPh in the DES at 300 K obtained from MD simulations. Experimental values for pure DES measured at 298.15 K from D'Agostino et al.[17]

Molecule	Diffusion coefficient $\times 10^{12}$ m^2 s^{-1}		
	pure DES	AcPh/DES	expt[17]
Ch^+	3.3 ± 0.2	3.7 ± 0.1	3.8
Cl^-	5.7 ± 0.1	5.6 ± 0.2	
Gly	5.6 ± 0.1	5.8 ± 0.2	5.2
AcPh		5.6 ± 0.8	

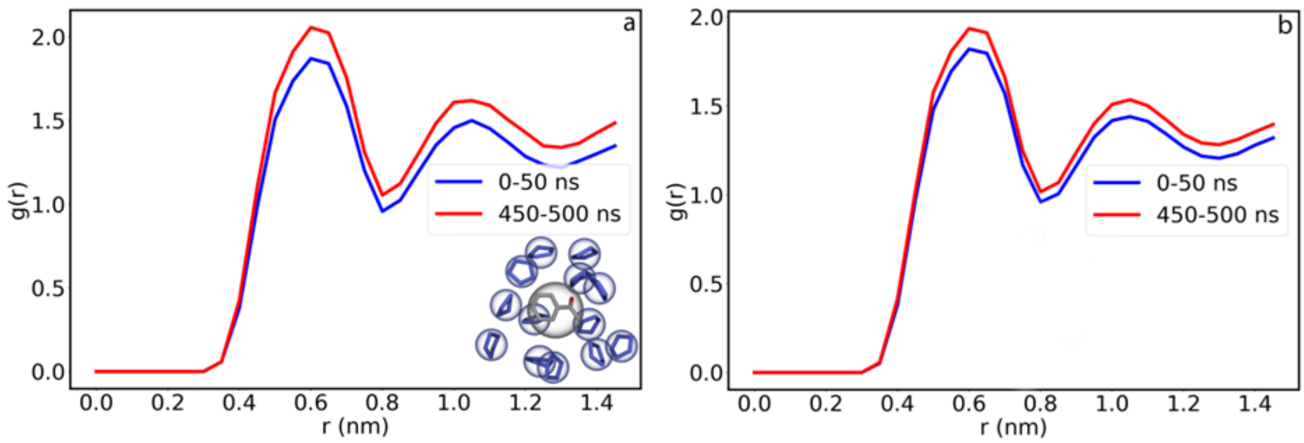

Figure S11 THF-AcPh centre of mass radial distribution functions at different simulation times for a 0.2 mmol/g (a) and 1.0 mmol/g solutions (b) in the DES/THF system.

The free energy of the transfer of AcPh from the deep eutectic solvent to THF was calculated using umbrella sampling simulations (see main text for the full description of the methodology). Computations showed a preference for AcPh for the organic phase with a difference of approximately 7.5 kJ mol^{-1}.

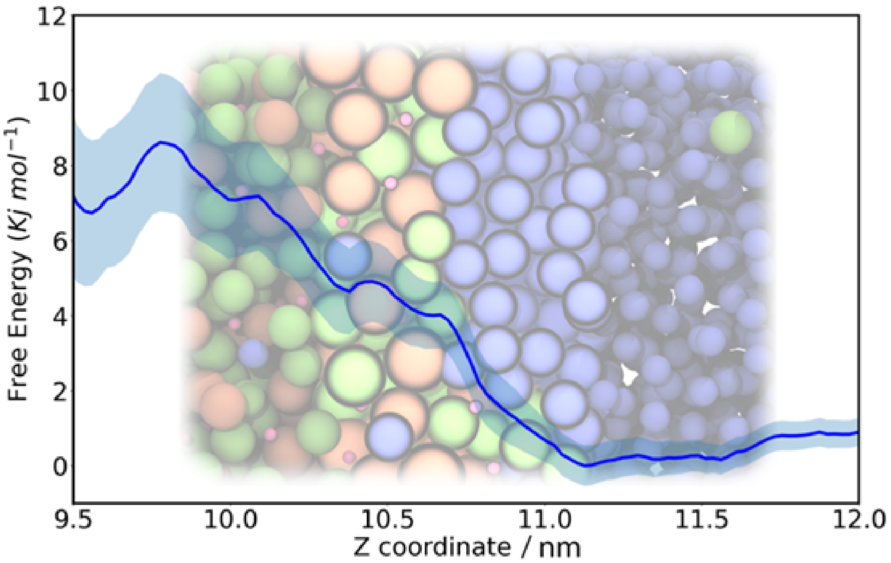

Figure S12 Free energy profile for the transfer of an AcPh molecule from DES to THF. The interface is represented by the highlighted beads and the blue shaded region the statistical uncertainty.

Simulation Details

The modelling study was conducted employing classical all-atom molecular dynamics (MD) in GROMACS 2023-dev.[18] Atomic charges and force field parameters for Gly and ChCl were taken from the literature,[19] whereas for THF a RESP fit of HF/6-31G* electrostatic potential provided the atomic charges and the standard GAFF[20] parameters were used for bonded interactions. The DES systems were modelled as a 1:2 ChCl:Gly solution in cubic 60 Å boxes, containing 800 Gly and 400 ChCl molecules. The AcPh solution in pure DES at a concentration of 0.2 mmol g^{-1} was obtained adding 26 AcPh molecules to the above-mentioned system. The interface system was modelled as a rectangular box of 60 × 60 × 250 Å in which the DES components (1668 Gly molecules and 834 ChCl molecules) were placed in the first half of the box along the Z axis and the rest was completed by 3337 THF molecules. Two solutions of AcPh in the DES/THF systems were modelled: one at a concentration of 0.2 mmol g^{-1} (adding 102 molecules of AcPh randomly distributed between the two phases) and a second at 1.0 mmol g^{-1} (adding 511 molecules of AcPh randomly distributed between the two phases). Finally, a system containing 54 molecules of AcPh and 54 molecules of iPrMgCl distributed in the DES and THF phases respectively was created to mimic a complete organometallic reaction environment. After steepest-descent energy minimization with a maximum force tolerance threshold of 200 kJ mol^{-1} nm^{-1}, molecular dynamics simulations were conducted in the NPT ensemble at 300 K using a stochastic velocity and box rescaling algorithm.[21] Bonds involving H atoms were constrained using the LINCS algorithm[22] and the selected timestep was 2 fs. For the mixed DES/THF systems, an initial 1 ns equilibration in which a semi-isotropic barostat was employed, accounted for the relaxation of the surface tension between the two immiscible phases. Due to the high viscosity of the DES a 10 ns annealing procedure was carried out, going

from 300 to 500 K in 1 ns cycles, followed by 500 ns of production simulations for the pure Gly, pure DES and AcPh solutions in DES. In case of the mixed DES/THF systems where both AcPh and iPrMgCl are present, production runs were extended to 1000 ns. Diffusion coefficients were calculated starting from mean squared displacements from 10 to 100 ns after checking that the system reached the diffusive regime in that window.[23] The systems containing only DES and DES plus AcPh were simulated for additional 50 ns at constant volume, after increasing the simulation box in the Z direction by a factor of 6, to simulate the DES/air interface. To ensure the equilibration of these systems, for the first 25 ns of the NVT simulation an annealing procedure was used taking the temperature from 300 to 500 K and back in 5 ns cycles.

Umbrella sampling simulations

Umbrella sampling (US)[24] was used to study the transfer of AcPh from the DES to the organic phase. The windows for US were prepared by extracting 21 snapshots from a short simulation in which a harmonic potential of 2000 $\text{kJ mol}^{-1}\,\text{nm}^{-1}$ was applied between the centre of mass of a specific AcPh molecule and that of the choline residues. The extracted snapshots were selected to span a range of positions that went from DES bulk to THF bulk. The applied force constant in each of the windows was 200 $\text{kJ mol}^{-1}\,\text{nm}^{-1}$ and a simulation time of 30 ns in the NPT ensemble at 300 K and 1 atm with was chosen to allow an exhaustive sampling of the bulk DES windows. Free energy profiles were obtained through the Weighted Histogram Analysis Method (WHAM) as implemented in the GROMACS package,[18] discarding the first 6 ps of each window.

References

[1] D. T. Bowron, A. K. Soper, K. Jones, S. Ansell, S. Birch, J. Norris, L. Perrott, D. Riedel, N. J. Rhodes, S. R. Wakefield, A. Botti, M.-A. Ricci, F. Grazzi, M. Zoppi, *Rev. Sci. Instrum.* **2010**, *81*, 033905.

[2] K. J. Edler, R. Atri, J. D. Holbrey, D. T. Bowron, O. S. Hammond, J. Hooton, E. Hevia, Deep Eutectic Solvents in Chemical Synthesis: Probing Solvent-Substrate Interactions, 10.5286/ISIS.E.99690292 **2018**.

[3] A. K. Soper, *GudrunN and GudrunX: Programs for correcting raw neutron and X-ray diffraction data to differential scattering cross section*, Science & Technology Facilities Council Swindon, UK **2011**.

[4] A. K. Soper, *Mol. Phys.* **2009**, *107*, 1667.

[5] A. K. Soper, *Chem. Phys.* **1996**, *202*, 295.

[6] A. K. Soper, *Mol. Phys.* **2001**, *99*, 1503.

[7] A. K. Soper, *Phys. Rev. B* **2005**, *72*, 104204.

[8] J. D. Berry, M. J. Neeson, R. R. Dagastine, D. Y. Chan, R. F. Tabor, *Jour. Colloid Interf. Sci.* **2015**, *454*, 226.

[9] R. Dalgliesh, S. Langridge, J. Plomp, V. de Haan, A. van Well, *Physica B* **2011**, *406*, 2346, proceedings of the 8th International Workshop on Polarised Neutrons for Condensed Matter Investigation.

[10] K. J. Edler, G. M. Neville, M. Campana, E. K. Bathke, I. Manasi, N. Leaman, T. Arnold, J. Hooton, Interfacial Chemical Reactions in Deep Eutectic Solvents, STFC ISIS Neutron and Muon Source, 10.5286/ISIS.E.RB2010710-2 **2021**.

[11] J. B. Brzoska, I. B. Azouz, F. Rondelez, *Langmuir* **1994**, *10*, 4367.

[12] A. Hughes, RasCAL, https://github.com/arwelHughes/RasCAL_2019/.

[13] P. Mark, L. Nilsson, *J. Phys. Chem. A* **2001**, *105*, 9954.

[14] A. K. Soper, *Int. Schol. Res. Notices* **2013**, *2013*, 279463.

[15] A. H. Turner, J. D. Holbrey, *Phys. Chem. Chem. Phys.* **2019**, *21*, 21782.

[16] Névot, L., Croce, P., *Rev. Phys. Appl. (Paris)* **1980**, *15*, 761.

[17] C. D'Agostino, R. C. Harris, A. P. Abbott, L. F. Gladden, M. D. Mantle, *Phys. Chem. Chem. Phys.* **2011**, *13*, 21383.

[18] M. J. Abraham, T. Murtola, R. Schulz, S. Páll, J. C. Smith, B. Hess, E. Lindahl, *SoftwareX* **2015**, *1-2*, 19.

[19] S. L. Perkins, P. Painter, C. M. Colina, *J. Chem. Engin. Data* **2014**, *59*, 3652.

[20] J. Wang, R. M. Wolf, J. W. Caldwell, P. A. Kollman, D. A. Case, *J. Comput. Chem.* **2004**, *25*, 1157.

[21] G. Bussi, D. Donadio, M. Parrinello, *J. Chem. Phys.* **2007**, *126*, 014101.

[22] B. Hess, H. Bekker, H. J. C. Berendsen, J. G. E. M. Fraaije, *J. Comput. Chem.* **1997**, *18*, 1463.

[23] M. G. Del Pópolo, G. A. Voth, *J. Phys. Chem. B* **2004**, *108*, 1744.

[24] G. Torrie, J. Valleau, *J. Comput. Phys.* **1977**, *23*, 187.
